# NALCN Ion Channels Have Alternative Selectivity Filters Resembling Calcium Channels or Sodium Channels

**DOI:** 10.1371/journal.pone.0055088

**Published:** 2013-01-28

**Authors:** Adriano Senatore, Arnaud Monteil, Jan van Minnen, August B. Smit, J. David Spafford

**Affiliations:** 1 Department of Biology, University of Waterloo, Waterloo, Canada; 2 Institut de Génomique Fonctionnelle, Centre National de la Recherche Scientifique, Montpellier, France; 3 Institut National de la Santé et de la Recherche Médicale, Montpellier, France; 4 Universités Montpellier I & II, Montpellier, France; 5 Hotchkiss Brain Institute, Faculty of Medicine, University of Calgary, Alberta, Canada; 6 Department of Molecular & Cellular Neurobiology, Center for Neurogenomics and Cognitive Research, Neuroscience Campus Amsterdam, Vrije Universiteit, Amsterdam, The Netherlands; Virginia Commonwealth University, United States of America

## Abstract

NALCN is a member of the family of ion channels with four homologous, repeat domains that include voltage-gated calcium and sodium channels. NALCN is a highly conserved gene from simple, extant multicellular organisms without nervous systems such as sponges and placozoans and mostly remains a single gene compared to the calcium and sodium channels which diversified into twenty genes in humans. The single NALCN gene has alternatively-spliced exons at exons 15 or exon 31 that splices in novel selectivity filter residues that resemble calcium channels (EEEE) or sodium channels (EKEE or EEKE). NALCN channels with alternative calcium, (EEEE) and sodium, (EKEE or EEKE) -selective pores are conserved in simple bilaterally symmetrical animals like flatworms to non-chordate deuterostomes. The single NALCN gene is limited as a sodium channel with a lysine (K)-containing pore in vertebrates, but originally NALCN was a calcium-like channel, and evolved to operate as both a calcium channel and sodium channel for different roles in many invertebrates. Expression patterns of NALCN-EKEE in pond snail, *Lymnaea stagnalis* suggest roles for NALCN in secretion, with an abundant expression in brain, and an up-regulation in secretory organs of sexually-mature adults such as albumen gland and prostate. NALCN-EEEE is equally abundant as NALCN-EKEE in snails, but is greater expressed in heart and other muscle tissue, and 50% less expressed in the brain than NALCN-EKEE. Transfected snail NALCN-EEEE and NALCN-EKEE channel isoforms express in HEK-293T cells. We were not able to distinguish potential NALCN currents from background, non-selective leak conductances in HEK293T cells. Native leak currents without expressing NALCN genes in HEK-293T cells are NMDG^+^ impermeant and blockable with 10 µM Gd^3+^ ions and are indistinguishable from the hallmark currents ascribed to mammalian NALCN currents expressed *in vitro* by Lu *et al.* in Cell. 2007 Apr 20;129(2):371-83.

## Introduction

The first evidence for the existence of NALCN ion channels came from *Drosophila* geneticist Hermann Muller during his classical X-ray mutagenesis studies in the 1930s [1]. Seventy years later, researchers identified the mutation attributed to Muller's “*narrow abdomen*” allele [2], as a deletion of 9 nucleotides within the coding sequence of a unique homologue of four repeat domain (4-domain) voltage-gated sodium (Na_v_) and calcium (Ca_v_) channels, designated as NALCN or Na_VI_2.1 [3]–[6]. NALCN is a single-copy gene crucial for survival in mammals, where gene knockdown is lethal in mice within 24 hours after birth due to severely disrupted respiratory rhythms [4]. Hippocampal neurons have resting membrane potentials ∼10 mV more hyperpolarized in NALCN−/− mice [4], and the channel is considered to provide a resting Na^+^ conductance in wild type neurons, not just in mammals [4] but also in nematodes (*C. elegans*) [7]–[9], fruit fly (*Drosophila*) [2], [10] and snail (*Lymnaea*) [11]. Analyses of mutant invertebrates suggest that NALCN is linked to anesthetic sensitivity [12], locomotion [13], diurnal rhythms [2], [10], gap junction activity [7] and synaptic vesicle turnover [8]. There also seems to be a link between NALCN and ionic balance, since heterozygous NALCN knockout mice have significantly elevated serum sodium levels [14]. NALCN expresses in a brain complex which includes accessory proteins UNC-79 and UNC-80 [15], [16].

A key feature attributed to NALCN is its role gating Na^+^ ions in a voltage-independent manner, providing a leak conductance into cells at rest to drive membrane excitability [4], [11]. Ion selectivity of related 4-domain Ca_v_ channels has been defined by signature residues of the selectivity filter, contributed by a single pore-loop (P-loop) residue from each of four repeat domains (I–IV), forming a ring of negatively-charged glutamate residues (EEEE) in Ca_v_1 (L-type) and Ca_v_2 calcium channels [17]–[19], or EEDD in Ca_v_3 (T-type) channels [20]. Traditional Na_v_1 sodium channels bear a DEKA pore in the equivalent position, and mutagenesis studies indicate that the lysine (K) residue in Domain III is both necessary and sufficient to generate ion channels with high Na^+^ selectivity over Ca^2+^ ions [17]–[20]. Equivalent sodium-selective channels in motile jellyfish have a DKEA pore configuration where the lysine residue has migrated to Domain II from Domain III [21]. Jellyfish Na_v_ channels with DKEA pores [21] are Na^+^-selective [22] but mutagenesis studies suggest that they are slightly less selective for Na^+^ ions than are DEKA Na_v_ channels [23]. Mammalian NALCN channels have the hallmark lysine residue in Domain III, characteristic of Na_v_ channels with an EE**K**E pore [3]. As expected from its EEKE structure, expression studies suggest that mammalian NALCN is permeable to Na^+^ ions and other monovalent cations and also moderately permeable to divalent Ca^2+^ [4].

Our phylogenetic analyses indicate that NALCN may have expanded roles outside of serving as a Na^+^ leak conductance channel. The ancestral NALCN has a selectivity filter that more resembles Ca^2+^ channels (EEEE), and most invertebrates have two alternatively-spliced isoforms of NALCN, one with a standard selectivity filter that resembles Na_v_ channels (EE**K**E or E**K**EE), and a second isoform that resembles Ca_v_ channels (EEEE). Both the Ca^2+^ and Na^+^ channel isoforms of NALCN are abundantly expressed in snails, with unique expression profiles in different tissues. The two isoforms of *Lymnaea* NALCN express at detectable protein levels with specific antibodies when transfected into HEK-293T cells, and leak currents in NALCN-transfected cells can be blocked by replacement of external Na^+^ ions with impermeant NMDG^+^, or by application of 10 µm trivalent cation blocker Gd^3+^ as reported for rat NALCN [4]. We also find comparable leak currents in HEK-293T cells without co-expressing NALCN cDNAs. Leak currents in control HEK-293T cells are equally blocked by NMDG^+^ and Gd^3+^ suggesting that these compounds are not specific for cells expressing NALCN channels. It would have been attractive to confirm a potential calcium-selectivity for snail NALCN-EEEE channels and a cation selectivity of snail NALCN-EEKE channels. NALCN proteins readily express in HEK-293T cells with coexpressed accessory proteins (UNC-79, UNC-80) and SRC kinase, but we are not convinced yet that NALCN forms ion conducting channels *in vitro*.

## Results

### NALCN is an unusually conserved and short, 4-domain ion channel

**Figure 1:**
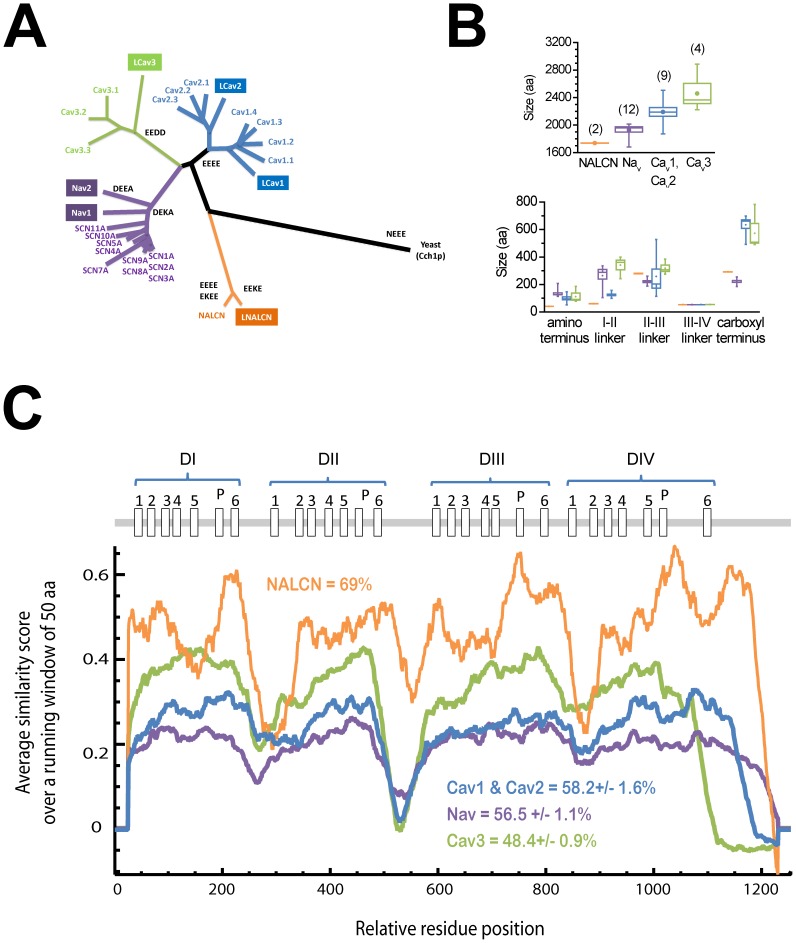
NALCN is unique amongst related 4-domain P-loop voltage-gated cation channels in it being a singleton gene in most reported genomes, and a coding region of small size and high conservation. Comparisons in panels A, B, and C are made between *Lymnaea* NALCN, LCa_v_1, LCa_v_2, LCa_v_3, Na_v_1, Na_v_2 and the 21 human homologs. (A) Gene tree of aligned sequences suggest a distant relationship to voltage-gated Ca_v_ and Na_v_ channels, and closer to the singleton yeast calcium channel. Grey letters indicate selectivity filter residues. (B) NALCN is a shorter than other cation channels in total size (top panel) largely because of its short amino terminus and I–II linker (bottom panel). Snail and human NALCN are within +/−3 aa of each other in sizes of individual domains and cytoplasmic linkers. (C) Running averages of similarity indicate that snail and human NALCN are much more conserved than other cation channels, with a high level of conservation even in the generally hypervariable cytoplasmic linkers and amino-termini.

NALCN is slightly closer in sequence to a yeast calcium channel [24] than to voltage-gated Na_v_ and Ca_v_ channels within the 4-domain ion channel family (**Figure 1a**), and has remained mostly a single gene, compared to the evolution of 20 different Ca_v_ and Na_v_ channel genes in vertebrates. Exceptions so far to the single NALCN gene in animals include sponge *Amphimedon*, anthozoan cnidarian *Nematostella*, and nematode *Caenorhabditis*, all of which have two genes. Snails, like most invertebrates, have three Ca_v_ channel genes, two Na_v_ genes and only one NALCN channel gene (**Figure 1a**). Comparing protein sizes between snails and humans, NALCN is almost invariant in size and smaller than most Na_v_ and Ca_v_ channels, with especially short amino termini and I–II linkers, and with invariant III–IV linkers that are 53 or 54 amino acids, common to all of these 4-domain channel types (**Figure 1b**). Comparisons between snail and human sequences illustrate a highly conserved NALCN structure across the whole protein (69% similarity), from the N-terminus to the C-terminus. The similarity between NALCN homologues is remarkable, given that similar comparisons between snail and human Na_v_ and Ca_v_ channels reveal more marked divergence (i.e. 48.4% and 58.2% respectively) (**Figure 1c**).

### NALCN genes can have alternatively-spliced pores

**Figure 2:**
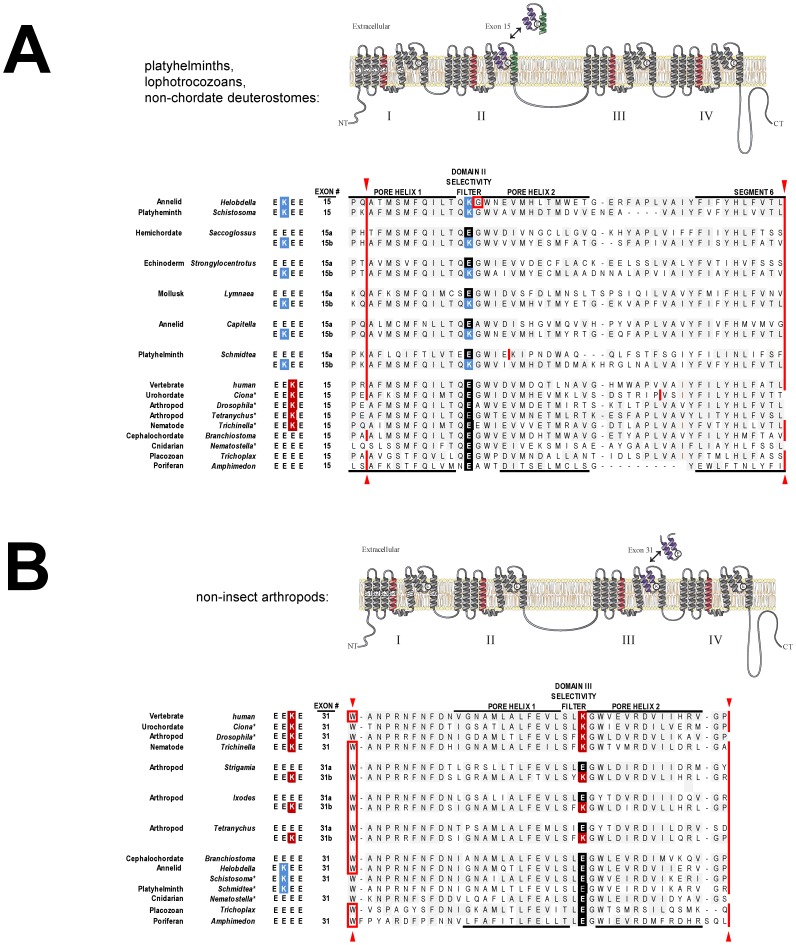
Representative NALCN sequences of alternative calcium- and sodium-selective pores generated by alternative exons (A) exon 15 (EEEE/EKEE) and (B) exon 31 (EEEE/EEKE). Exons 15 and exon 31 (delimited by splice junctions shown in red), span the re-entrant P-loop (pore helices surrounding the selectivity filter) in Domains II and III, respectively. Exon 15 also includes the proximate region of segment 6, while exon 31 includes the distal portion of the extracellular turret. Splice junctions for exons 15 and exon 31 are conserved in the earliest metazoans (sponge, placozoans), which are duplicated in most invertebrate phyla to produce tandem mutually-exclusive exons where the 5′ exon codes for exon ‘a’ with E in the selectivity filter (15a, 31a) and the 3′ exon codes for exon ‘b’ with K in the selectivity filter (15b, 31b). Either the donor or acceptor site of exon 15 or 31 are lost in most species outside chordates (indicated by *) that don't have alternative-splice junctions to generate both calcium and sodium selective pores. Exons 15a/15b have variable sequence between the pore and segment 6 for generating a gene tree (**see** **Figure 4**).

In light of the relative invariance of NALCN sequences, it is remarkable that we identified alternative splicing within a pore domain when cloning the full length homolog from the pond snail *Lymnaea stagnalis*. Alternative exons 15a and 15b span the re-entrant pore helices and selectivity filter residues to the proximate end of segment 6 in Domain II (**Figure 2a**). The obvious difference between these exons is the key selectivity filter residue for ion permeation, located at the most constrictive point of the “hourglass” pore as evidenced by the three-dimensional structure of the prokaryotic sodium channel Na_v_Ab [25]. The resulting selectivity filter motifs for snail NALCN are E**E**EE (with exon 15a) and E**K**EE (with exon 15b) (**Figure 2a**). Mining of NALCN genes from other invertebrate genomes revealed homologous alternative exons 15a and 15b, with an upstream exon 15a bearing a glutamate (E) in the selectivity filter, and a downstream exon 15b coding for lysine (K) in this position (**Figure 2a**). Dual E**E**EE/E**K**EE pores are present in the genomes of species of platyhelminths (flatworms), and lophotrochozoan protostomes (mollusks and annelids), and non-chordate deuterostomes (echinoderms and hemichordates) (**See Appendix S1 for a listing of genomic sequences spanning exon 15**). Similarly, exon 31 is alternatively-spliced in what is likely a completely separate evolutionary event, coding for the pore in Domain III instead of Domain II, with an upstream exon 31a with glutamate (E) in the selectivity filter, and a downstream exon 31b coding for lysine (K) within the arthropods (i.e. EE**E**E/EE**K**E), including species of Myriapod (e.g. centipedes) and Chelicerates (mites and ticks) (**Figure 2b**) (**See Appendix S2 for a listing of genomic sequences spanning exon 31**). The only animal groups that don't appear to contain species with alternative (EEEE) and (E**K**EE/EE**K**E) pores for NALCN, coincidentally, include the species most often used as animal models, including nematodes (i.e. *C. elegans*), insects (*Drosophila*) and chordates (rat and mouse).

### Patterns of evolution of NALCN's alternatively-spliced pores

**Figure 3:**
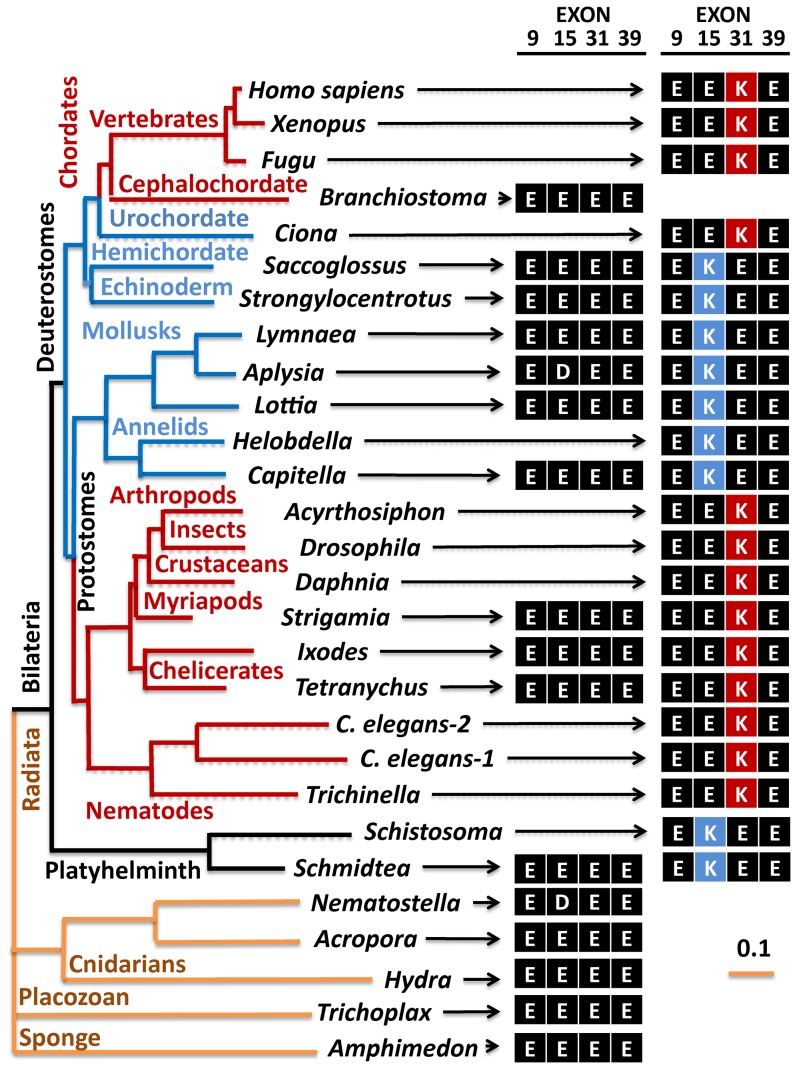
Gene tree illustrates that the NALCN gene is conserved in reported metazoan genomes with alternative selectivity filters that generate a choice of calcium-selective and sodium-selective pore configurations in invertebrates. NALCN first appears with a selectivity filter resembling a calcium-selective pore (EDEE or EEEE) in the earliest metazoans (sponges and placozoans) with a simple body plan. A duplication of Exon 15 coding for the selectivity filter residue in Domain II creates alternative calcium-selective (EEEE) and sodium-selective (EKEE) pores in the early nervous systems of Bilateria, including platyhelminthes, the planarian (*Schmidtea*) and protostomes of the lophotrochozoan lineage (mollusks and annelids), and non-chordate deuterostomes (echinoderms and hemichordates). A duplication event in Exon 31 of the Ecdysozoan lineage creates different alternative calcium-selective (EEEE) and sodium-selective (EEKE) pores that are retained in myriapods (includes centipedes, millipedes) and chelicerates (includes Arachnids like mites and ticks). The cephalochordate genome (*Branchiostomia*, amphioxus) only has a calcium-selective pore (EEEE), and chordates (urochordates, vertebrates) are the only lineage to lack a calcium-selective pore having only sodium-selective pores (EEKE). Dashed lines indicate a likely loss of an alternative exon 15 or 31.

**Figure 4:**
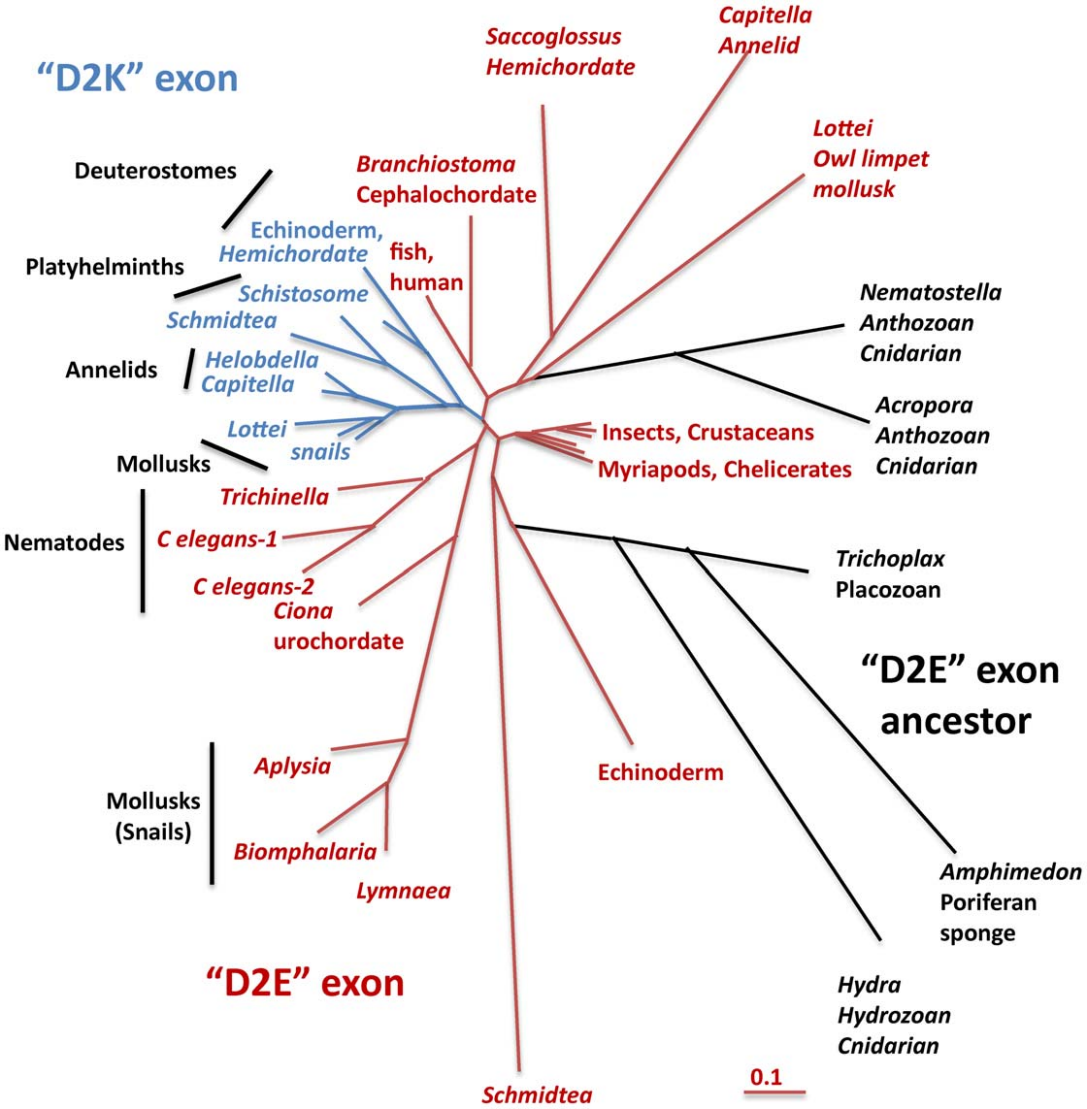
Gene tree of exons 15a/15b indicates that tandem exon 15b, coding for Lys (K) in the selectivity filter, is monophyletic. Exon 15b likely duplicated from exon 15a that codes for Glu (E) in the selectivity filter from an ancestor of early metazoans with a single exon 15a (sponge, *Trichoplax*, cnidarian), creating an exon 15b with Lys (K). The common root of exon 15b suggests that the dual, mutually-exclusive exons 15a (E) and 15b (K) originated in early metazoans (represented in planarian *Schmidtea*, a Platyhelminth), evolving once to be subsequently retained in lophotrochozoans (annelids, mollusks) and hemichordates, before being lost in chordates. There was a secondary loss of exon 15a in some invertebrates such as platyhelminth (*Schistosoma*) and annelid (*Helobdella*). No NALCN gene codes for EKKA, so exon 31b (with a K) likely evolved from exon 31a in an arthropod ancestor with an EEEE selectivity filter common to myriapods and chelicerates, with exon 31a being secondarily lost in insects and crustaceans. Exon 31 is shorter and more highly conserved than exon 15, only spanning some of the extracellular turret and the downstream selectivity filter. Exon 31a and 31b barely differ outside of the single E and K residue in the selectivity filter suggesting that exon 31b (K) could have had a separate evolution from an EEEE ancestor in nematodes and chordates.

The simplest multicellular organisms (sponge, *Trichoplax* and cnidarians) lack a Na^+^-like selectivity filter with a lysine residue (E**K**EE or EE**K**E) (**Figure 2** **,** **Figure 3**), and thus it is likely that the ancestral NALCN was Ca^2+^ channel-like with an EEEE (or EDEE) pore. We can also suppose that the EKEE pore evolved from an exon duplication of a NALCN Ca^2+^ channel (EEEE pore) in a common ancestor to all species with an EKEE pore, including platyhelminths (flatworm), mollusks, annelids, echinoderms and hemichordates (acorn worm). Alternate exon 15 has variable sequences between pore helix 2 and segment 6 that enable analysis of a proposed evolution of exons 15a and 15b (**Figure 2a**). All 15b exons cluster together, appearing monophyletic in a gene tree of exon 15a and exon 15b sequences (**Figure 4**). Alternate exons 31a and 31b are delimited to the highly conserved pore helices, and are highly similar to one another except for the pore selective ‘E’ or ‘K’ residue, which prevents assessment of their evolutionary history (**Figure 2b**). Interestingly, alternative exons 31a and 31b are organized in genomes in a similar pattern as are exons 15a/15b, where exon ‘a’ with pore residue ‘E’ precedes the exon ‘b’ with pore residue ‘K’. Animals with alternative EEEE and EEKE pores likely arose once, in a common ancestor because animals with these dual pores are clustered within a lineage of Arthropods (**Figure 3**). Insects and crustaceans, and more distantly related animals, such as nematodes, urochordates and vertebrates only have a NALCN with an EEKE pore, which could each have arisen independently from an ancestral EEEE isoform.

### Expression patterns of alternatively-spliced NALCN pores

**Figure 5:**
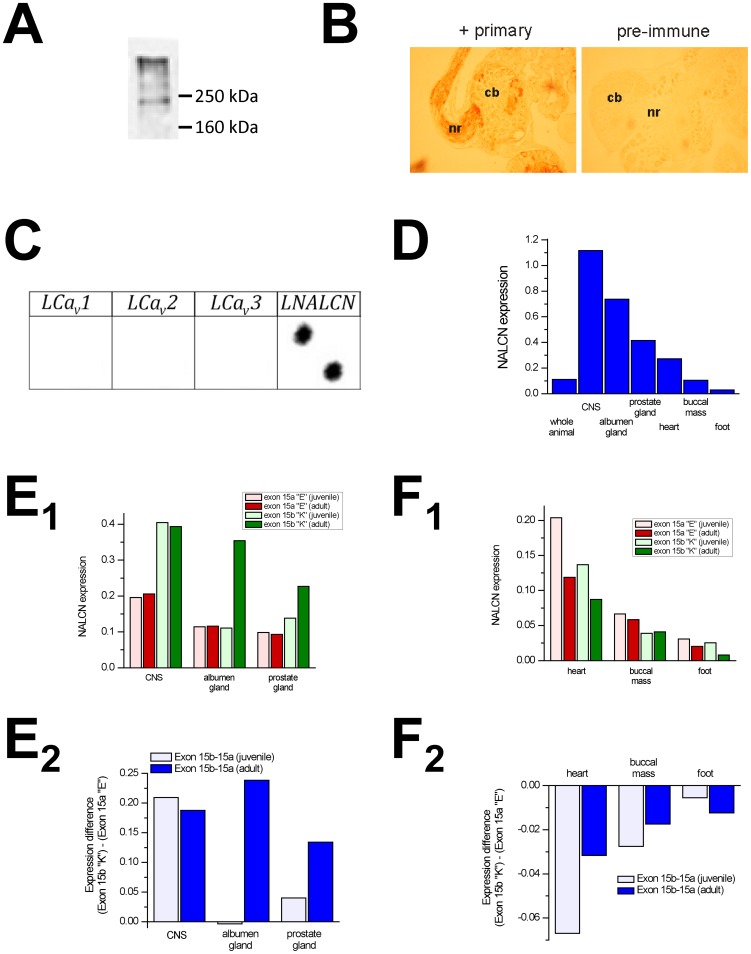
NALCN mRNA expression in *Lymnaea* pond snail measured by quantitative PCR reveals unique expression profiles of sodium-selective pore (EKEE) and calcium-selective pore (EEEE) variants. (A) *Lymnaea* NALCN antibody identifies appropriately-sized ∼200 kDa NALCN protein in *Lymnaea* brain homogenate of a Western blot. (B) Antibody staining of *Lymnaea* brain using polyclonal antibodies reveals that NALCN protein is enriched in neurites (nr) protruding from cell bodies (cb). (C) Reverse-northern blotting reveals that the *Lymnaea* NALCN mRNAs specifically accumulate in neurites/axons. (D) NALCN is more abundantly expressed in secretory organs (brain, albumen gland and prostate) than muscle (heart, buccal mass and foot). (E_1_) Expression of EKEE-NALCN is twice as abundant in the brain than EEEE-NALCN, and EKEE specifically rises in reproductive organs (albumen gland and prostate) during sexual maturation of juveniles to adult animals. (E_2_) The substantially higher expression of EKEE-NALCN versus EEEE-NALCN in the brain and reproductive organs of sexual mature snails, suggest that the EKEE-NALCN isoform is associated with secretion. (F_1_) NALCN expression falls in heart, buccal mass and foot from juvenile to adult. (F_2_) There are greater expression levels of EEEE-NALCN in heart, buccal mass and foot compared to EKEE-NALCN, suggesting that EEEE-NALCN has greater functions associated with muscle (heart, buccal mass, foot).

Polyclonal antibody raised against snail NALCN sequence identify a NALCN-sized protein in Western blots from snail brain homogenate (**Figure 5a**), and label in a dense-staining pattern along a particular subset of snail neurons identified in brain sections (**Figure 5b**) that is consistent with NALCN staining patterns within axons of the *C. elegans* nervous system [9]. Additionally, we find that blots spotted with cDNA coding for 760 bp of the NALCN gene in the C-terminus, but not related snail Ca_v_ channels genes, hybridize with a probe consisting of cDNA generated from mRNA of isolated snail axons (**Figure 5c**), suggesting localization of NALCN mRNA within axons. The dense immuno-staining of the brain is consistent with the general expression pattern of snail NALCN measured by quantitative RT-PCR, which is highest in the brain, followed by secretory glands (albumen and prostate), then heart, followed by muscle (buccal mass and foot) (**Figure 5d**). Isoform-specific primers indicate that the expression of NALCN-EKEE is 2× greater than NALCN-EEEE in the brain, and only NALCN-EKEE upregulates from juvenile to adult snails in the brain and in albumen and prostate glands that mature into secretory organs **(** **Figure 5e_1_**, **Figure 5e_2_**). NALCN-EEEE is 2× less abundant than NALCN-EKEE in the brain, while NALCN-EEEE expresses more in the heart (40 to 50% more) and also muscle, such as buccal mass (40 to 70% more) compared to NALCN-EKEE (**Figure 5f_1_**, **Figure 5f_2_**). The differences between the tissue expression profiles of NALCN-EEEE and NALCN-EKEE suggest that they are associated with different functions in different cell types, which likely depend solely on their different selectivity filters, since the remainder of the channel protein is lacking in alternative splicing (**see Materials and Methods**).

### In vitro expression and electrophysiological recording of the snail NALCN isoforms

**Figure 6:**
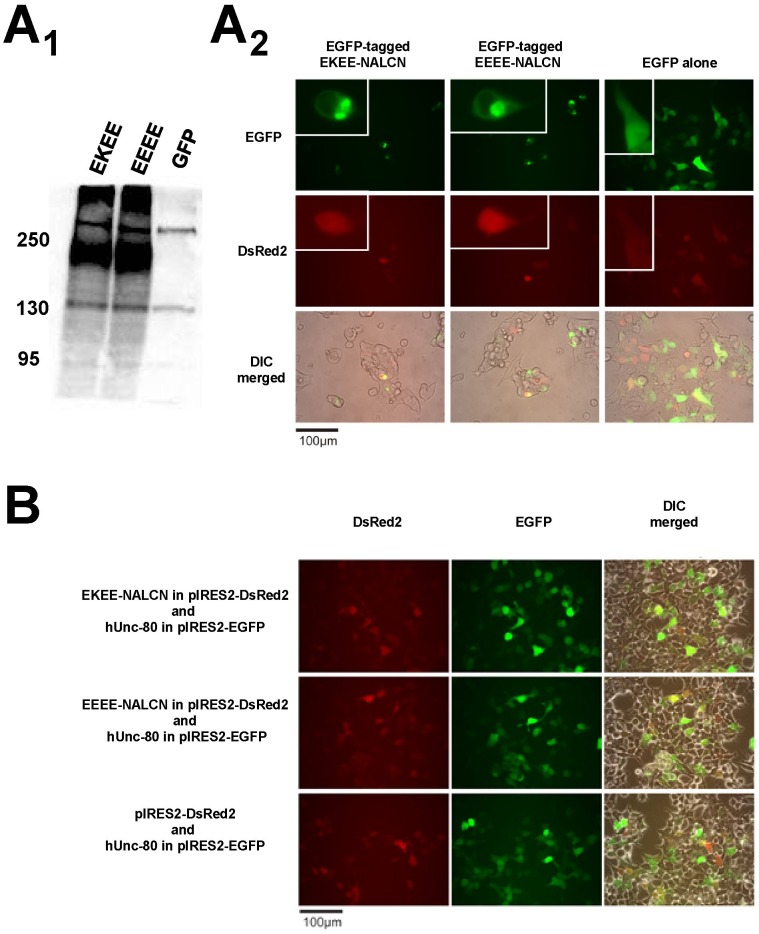
*Lymnaea* EKEE-NALCN and EEEE-NALCN isoforms express in the endomembrane system of HEK-293T cells, and co-express with key auxiliary subunit human Unc-80. (A_1_) EGFP-coupled *Lymnaea* NALCN cDNAs express in HEK-293T cells as appropriately-sized proteins (EGFP = 27 kDa, *Lymnaea* NALCNs = 200 kDa) on Western blots detected by GFP antibody (Amsbio, Lake Forest, CA, USA). Western blot banding pattern of transfected EGFP alone is shown for comparison. (A_2_) EGFP-coupled *Lymnaea* NALCN isoforms appear as membrane-delimited staining (inset) compared to more generalized staining resulting from co-transfected DsRed2. (B) *Lymnaea* NALCN and human Unc-80 co-transfected in bicistronic pIRES2 vectors indicate that the abundance of NALCN isoform expression (DsRed2 label) correlates with the abundance of expressed hUnc-80 (EGFP label). Human Unc-80 expression does not co-relate with DsRed2 expression lacking NALCN cDNAs on the pIRES2 vector.

Enhanced green fluorescent protein (EGFP)-tagged snail EEEE and EKEE NALCN isoforms are identifiable as expressed proteins on Western blots of transfected HEK-293T cells at the appropriate size (EGFP  = 27 kDa, snail NALCN = ∼200 kDa) using anti-GFP antibodies (**Figure 6a_1_**). Transfected, GFP-tagged snail NALCN isoforms have a more localized staining in HEK-293T cells (**Figure 6a_2_**), compared to the relatively generalized fluorescence of co-expressed red fluorescent protein DsRed2. We also co-expressed Unc-80, which serves as a key accessory subunit for NALCN expression and function in invertebrates [8], [9] and mammals [15], [16]. Positively-transfected cells expressing snail EEEE and EKEE NALCN isoforms were identified by DsRed2 fluorescence, using NALCN cloned into bicistronic vector pIRES2-DsRed2 (**Figure 6b**). DsRed2-positive, NALCN-expressing cells corresponded to the same cells that were EGFP-positive, co-transfected with human Unc-80 in bicistronic vector pIRES2-EGFP (**Figure 6b**). DsRed2-expressing cells did not correspond to EGFP-positive, Unc-80 expressing cells, when pIRES2-DsRed2 was transfected without the snail NALCN cDNA in the bicistronic vector (**Figure 6b**). While Unc-80 expression seems to correlate with that of snail NALCN when co-transfected into HEK-293T (**Figure 6b**), Unc-80 is not required for snail NALCN expression in these cells (**Figure 6A_1_**, **Figure 6A_2_**).

**Figure 7:**
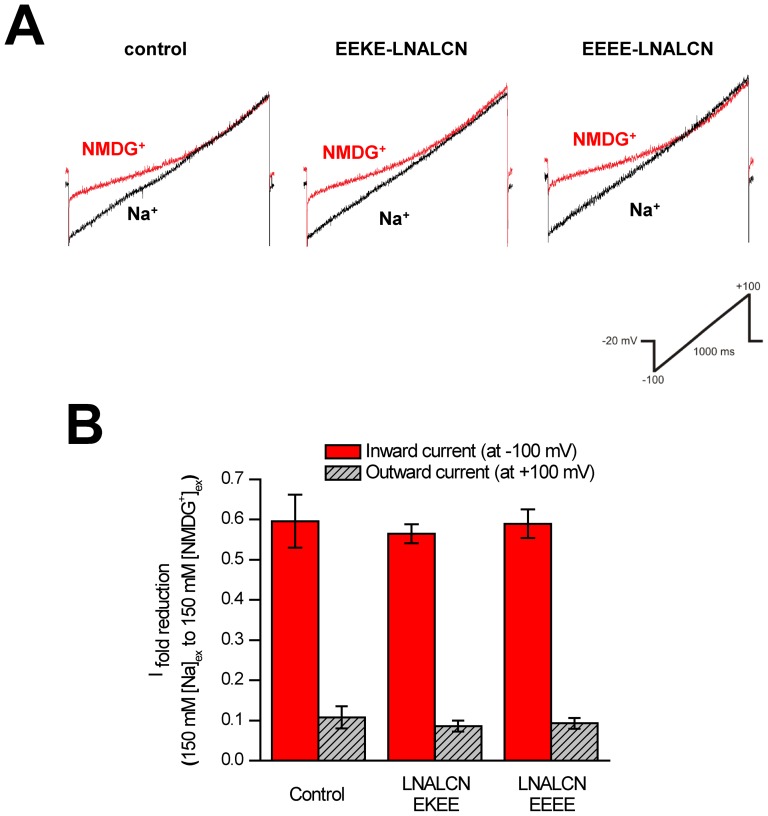
Leak currents from snail NALCN-expressing HEK-293T cells were indistinguishable from those in control cells lacking NALCN. (A) Sample traces showing the block of inward current when replacing 150 mM external sodium (black bars) with NMDG^+^ (hatched). HEK-293T cells were co-transfected with either 1 µg of *Lymnaea* NALCN pore variants (EKEE or EEEE) in bicistronic pIRES2-DsRed2, or empty pIRES2-DsRed2 vector for control, plus 1 µg of human Unc-80 in pIRES2-EGFP (enhanced green fluorescent protein), and 1 µg of a constitutively active SRC kinase (Y529F) in a pUSEamp vector. Leak currents, recorded under whole-cell voltage clamp using a ramp protocol (inset), were observed under all conditions. (B) A similar reduction in both inward and outward currents (i.e. at -100 mV and +100 mV respectively) was observed when 150 mM external sodium was replaced with impermeant cation NMDG^+^ via perfusion (control n = 5; EKEE n = 7; EEEE n = 4).

**Figure 8:**
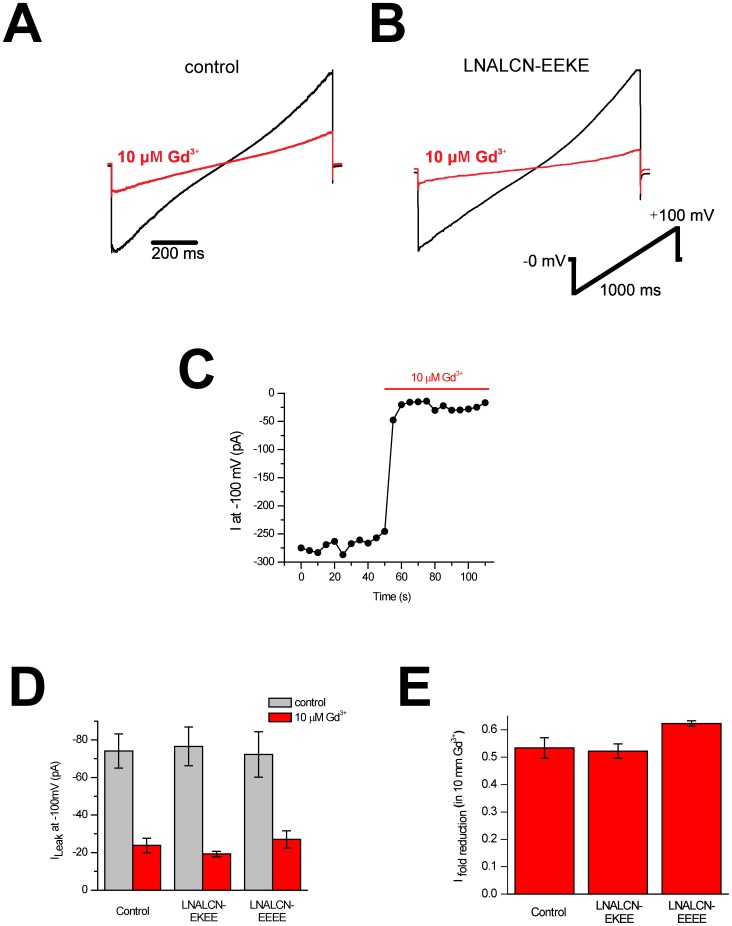
Non-specific leak ion currents are as sensitive to trivalent blocker Gd^3+^in HEK-293T cells as control cells lacking expressed NALCN gene. (A,B) Representative traces of steady state leak currents cells (A) expressing NALCN-EKEE or (B) control cells before (black) and after (red) application of 10 µM Gd^3+^ elicited by the ramp protocol shown and recorded using whole cell patch clamp of HEK-293T cells. (C) Plot showing the rapid Gd^3+^ block of leak current at −100 mV in a control cell. Wash-out was slow and in some cases the Gd^3+^ block persisted after washout.(D) Average ionic current size at −100 mV of HEK293T cells expressing the two isoforms of *Lymnaea* NALCN (EKEE, n = 13 or EEEE, n = 12). NALCN expressing cells are indistinguishable from currents of control cells transfected with empty vector pIRES2-DsRed2 (n = 16, black bars; all cells were co-transfected with 8 µg of channel/control vector, 8 µg of human Unc-80 in pIRES2-EGFP vector, and 0.5 µg of vector expressing a constitutively active SRC kinase). External perfusion of 10 µM Gd^3+^ led to a similar reduction in leak currents in all conditions (hatched bars). (E) Fold reduction in inward current at −100 mV upon application of 10 µM external Gd^3+^ for the same cells in A.

We can measure leak conductance currents in HEK-293T cells when EEEE- and NALCN-EKEE isoforms are co-expressed with auxiliary subunits of NALCN (Unc-80 and a constitutively active SRC kinase), that are expected to enhance NALCN expression *in vitro* [15], [16] (**Figure 7**, **Figure 8**). However, we also record the same Na^+^ leak currents in our HEK-293T cells, impermeant to NMDG^+^ ions, when we transfect Unc-80 and SRC without transfecting NALCN constructs at all (**Figure 7**). Notably, HEK-293T cells do not express endogenous NALCN channels [6] but have native leak conductances that are indistinguishable from those reported for NALCN [4]. We tested trivalent cation blocker gadolinium (Gd^3+^), reported to be a specific blocker of NALCN currents in HEK-293T cells [4]. 10 µM Gd^3+^ equally blocks leak currents in our transfected cells without NALCN expression (53.4±3.7%) in the same manner as cells transfected with *Lymnaea* NALCN constructs (**Figure 8a**, **Figure 8b**), with Gd^3+^ blockade for NALCN-EKEE of 52.2±2.6% and 62.3±1.0% block for NALCN-EEEE (**Figure 8d** **,** **Figure 8e**). We replicate the profile of drug block in control cells with Gd^3+^ blockade which eclipses 80% block within seconds after onset of drug perfusion (**Figure 8c**). Washing out Gd^3+^ is slow and difficult. Despite our best efforts, we were unable to ascribe selectivity differences between the unique pore isoforms of expressible *Lymnaea* NALCN transcripts, corroborating with others the difficulties in assessing leak currents from transfected NALCN genes in mammalian cells [3], [6], [9]. Alternative splicing of *Lymnaea* NALCN, that alters the selectivity filter residues and is thus expected to dramatically influence ion selectivity, will be evaluated in future studies, but outside of mammalian cell lines such as HEK-293T which possess native sodium leak currents that are indistinguishable from those reported from NALCN-transfected cells [4].

## Discussion

NALCN has been described as a Na^+^ leak conductance channel which contributes to membrane excitability and rhythmic behaviors [2], [4], [9], [11]. Here we report that the snail NALCN homolog has alternatively-spliced isoforms that generate a selectivity filter that resembles a Ca^2+^-selective channel as well as a non-specific or Na^+^-selective channel. The selectivity filter is exquisitely structured to define the selectivity of ions that interact with and permeate though the pore, and as such, alterations by alternative splicing, are expected to alter the relative affinity for the two major inward-permeating ions, Ca^2+^ and Na^+^. Both NALCN isoforms are abundantly expressed in snail tissues, and the alternative Ca^2+^ channel-like and Na^+^ channel-like pores of NALCN channels, evolved at least twice within completely different lineages of invertebrates, via alterations in Domains II and III. We look at 4×6TM channels across the animal kingdom to gain insights into the origin and function of NALCN channels with putative Ca^2+^ and Na^+^ selective pores.

### Evolution of 4×6TM channels

Four domain (4×6TM) ion channels likely evolved from single domain (1×6TM) ancestors such as those in prokaryotes, through duplication of domains and divergence of these domains. All 4×TM channels in animals have an invariant III–IV linker size that is 53 or 54 amino acids, a region in Na_v_ channels serving in a fast inactivation gating mechanism [26]. Na_v_ and Ca_v_ channels have more in common with each other than NALCN, and a shared genomic heritage with locations of shared intron splice sites, including rare AT-AC, U12-splice sites [27].

### Structure of 4×6TM channel pores

**Figure 9:**
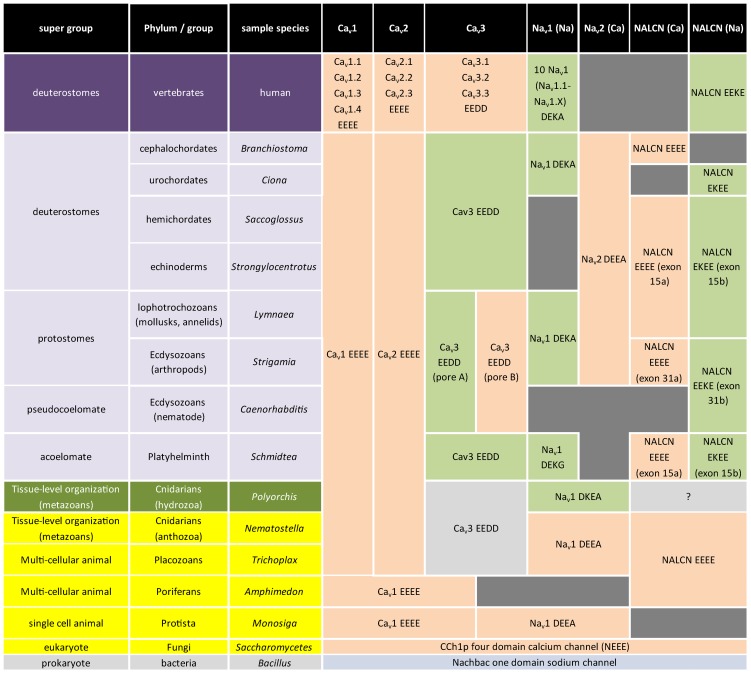
Conservation patterns suggest greater flexibility in calcium and sodium selectivity in 4-domain cation channels before vertebrate evolution. All 4-domain cation channels first existed with calcium-selective pores (i.e. Ca_v_ (EEEE), Na_v_ (DEEA) and NALCN (EEEE)). The first sodium-selective pores (DKEA) arose in extant relatives of the simplest animals with pelagic lifestyles and nervous systems (e.g. hydrozoan jellyfish) that produce sodium-dependent action potentials. Dual sodium- and calcium-selectivity evolved for all 4-domain channel types (excluding Ca_v_1 and Ca_v_2 channels) by different means. Traditional Na_v_ channels, such as the 10 vertebrate Na_v_ genes, have mostly DEKA selectivity filter pores (i.e. Na_v_1), with closely-related but more calcium-selective Na_v_2 channel genes with DEEA pores. Dual sodium- and calcium-selectivity also arose for NALCN and T-type channels, via alternative splicing of channel pores. In general, vertebrate ion channels becomes more exclusively sodium- or calcium-selective, with loss of a calcium-selective Na_v_2 gene, and loss of a NALCN splice isoform with the calcium selectivity filter (EEEE) and loss of T-type calcium channels with a capacity for sodium permeation.

A defining feature of each 4×6TM channels is its pore selectivity, largely governed by the P-loop which ascends to a most constrictive point of the “hourglass” pore where side chains of a critical residue contributed by each domain face into the pore (which is either E, D, K or A) between two highly conserved, pore helices (ten aa in length), demonstrated in the three-dimensional structure of the single domain, prokaryotic Na_v_ channel [25]. The symmetrical pore of this prokaryotic homomultimeric one domain channel has a glutamate (E) in this critical position, forming a Na^+^-selective channel. Mutagenesis of 4×6TM channels reveals that each re-entrant pore is not equal in its contribution as in homomultimeric channels, but instead, each side chain of the signature residues lies asymmetrically with respect to the plane of the conducting pore. The three lineages of 4×6TM channels (Ca_v_, Na_v_ and NALCN) appear to follow universal rules, almost without exception, in eight established pore configurations, including Ca^2+^-selective channels with negatively-charged glutamates and aspartates (EEEE, EDEE, EEDD, DEEA) and Na^+^-selective channels with a positively-charged lysine (K) in either the 2^nd^ or 3^rd^ domain (EKEE, EEKE, DKEA, DEKA) (**Figure 9**).

### Evolution of 4×6TM channel pores

The lineage of Ca_v_ channels have an EEEE pore for highest calcium selectivity, but Ds can replace Es; NALCN and Na_v_ channels have DxxA and ExxE pores, respectively, where xx = EE or EK or KE. Simplest organisms (choanoflagellates, sponges, placozoans and anthozoan cnidarians (coral, sea anemone) lack Na^+^-dependent action potentials and have pore configurations lacking an internal K residue (e.g. DEEA, EEEE) when cation channel genes are present in these organisms (**Figure 9**). The first appearance of animals with a nervous system and a pelagic adult lifestyle are the hydrozoan [21] and scyphozoan [28] jellyfish which are also the simplest organisms to have a lysine residue (K) in their Na_v_ channel (DKEA) (**Figure 9**). Within invertebrate groups, Na_v_ (DEEA/DKEA/DEKA) and NALCN (EEEE/EDEE/EKEE/EEKE) channels adopt multiple pore configurations with alternative selectivity for Ca^2+^ and Na^+^ ions, when K can appear in lieu of an internal E in Domains II or III. The dual Ca^2+^ or Na^+^ selectivity filters are lost in vertebrate NALCN and Na_v_ channels, and are restricted to EEKE and DEKA configurations, respectively which has the lysine residue in Domain III, positioned where it is expected to have the highest selectivity for Na ions, compared to the less selective EKEE and DKEA pores where the lysine residue is in Domain II instead, found in some invertebrates (**Figure 9**). The overall pattern of evolution suggest a primordial world of four domain channels gating Ca^2+^ ions, an experimentation and diversity of mixed Ca^2+^ and Na^+^ signalling in invertebrates, to a lack of Ca^2+^ signalling in vertebrate Na_v_ channels and NALCN (**Figure 9**).

### Parallel evolution of NALCN and Na_v_ channel pores

The co-appearance of NALCN and Na_v_ channels with a lysine residue in the pore is consistent with the evolution of Na^+^ ions to generate membrane excitability in nervous systems, circumventing the toxicity that intracellular Ca^2+^ ions have as agents for membrane depolarization [29]. There may also be a link between NALCN and Na_v_ channels to Na^+^ homeostasis, since many vertebrates with the lysine residue-containing pore are terrestrial vertebrates where Na^+^ retention and Na^+^ transport is especially critical. The presence of Ca^2+^-selective pores of NALCN and Na_v_ channels in invertebrates may relate to a more flexible signaling using Ca^2+^ and/or Na^+^ ions, and perhaps serving as an adaptation for invertebrates for the more variable Ca^2+^ levels in the aquatic environment and to service additional Ca^2+^ requirements in many invertebrates.

### The calcium-like EEEE NALCN pore with the configuration tends to be selectively lost in some species

The calcium-like NALCN-EEEE pore appears to be less essential because it is often selectively lost in individual species within phylogenetic groups containing the alternative EEEE and lysine containing EKEE or EEKE NALCN pore. Polychaete worms (*Capitella*) within the annelids retain both EEEE and EKEE forms of NALCN, but leech (*Helobdella*) retains only the EKEE isoform. Likewise, ectoparasitic tick (*Ixodes*) and spider mite (*Tetranychus*) have both NALCN-EEEE and NALCN-EEKE isoforms, but ectoparasitic mite (*Varroa*) retains only the EEKE isoform. Furthermore, EEEE and EEKE forms of NALCN and Na_v_ channels are present in non-parasitic flatworms such as the free-living planarian (*Schmidtea*), but the EEEE isoform of NALCN and Na_v_ channels are lost in closely-related trematodes, *Schistosoma* and *Clonorchis*. Endoparasitic flatworms cycle between living in the regulated environment within snails and vertebrates, perhaps where there is less need for Ca^2+^ and Na^+^ ion homeostasis, and requirements for rapid action potentials carried by Na^+^ ions and the retention of an EEEE form of NALCN and Na_v_ channels.

### All selectivity filter sequences of 4×TM channels fall into either Na^+^ or Ca^2+^-selective categories

The configuration of selectivity filter residues of every NALCN gene in more than a dozen animal phyla, abide by a set of rules that can be categorically identified as calcium-selective channels, with a calcium-like EDEE/EEEE pore, resembling the invariant EEEE calcium-selective filter of Ca_v_ channels and the DEEA configuration of calcium-permeable, invertebrate Na_v_2 channels. Or, categorically, the NALCN pore is consistent with a sodium-selective pore with an EKEE or EEKE configuration, resembling the DEKA or DKEA pores of Na_v_1 channels. NALCN pores parallel the pores of Na_v_ channels in evolution, with NALCN and Na_v_2 channels bearing primordial EEEE/DEEA calcium channel-like pores that become more sodium selective in invertebrates. While the phylogenetic data is highly consistent with NALCN pores with calcium (EDEE/EEEE) or sodium (EKEE/EEKE) selectivity, we were unable to confirm this by *in vitro* expression.

### NALCN is highly invariant and mostly a single gene in most species

Plasma membrane-associated ion channels are noted for an almost universal explosion in the duplication of genes, which for 4×6TM channels increase in number from five channel genes (three Ca_v_, two Na_v_, and one NALCN) in invertebrates to 21 vertebrate channel genes (ten Ca_v_, ten Na_v_, and one NALCN). Increasing gene numbers in Ca_v_ and Na_v_ channels endows novel adaptations in different tissue environments such as brain, heart, or skeletal muscle [30]. Other membrane-associated ion channels also have large numbers of genes such as Trp channels (27 genes) [31] or two pore, K^+^ leak channels (11 genes) [32]. NALCN is unusual in remaining a single gene, expressing a relatively short and highly conserved protein end to end, outside of the variability in the pore. NALCN has resisted expansion of gene numbers from multicellular organisms like sponges which lack highly-specialized cells, to humans, associated with complex tissue evolution, such as the brain, where NALCN is most abundant [33].

### NALCN as a calcium and/or sodium sensor

A perceived interchangeability of Ca^2+^ and Na^+^ selectivity in NALCN channels of this otherwise exceptionally conserved gene, is hard to reconcile given the very different roles that these ions play in excitability, where the relatively inert Na^+^ is much more abundant than Ca^2+^ and serves mostly in an electrogenic role, while Ca^2+^ is maintained at very low levels in cells due to cytotoxicity, and serves as an exquisitely-sensitive signalling molecule [29]. It is conceivable that NALCN is a specialized receptor for calcium or sodium ions, but not always permeable as a typical membrane-associated channel, such as the Ca_v_1.1 channel that has specialized in vertebrates as a calcium-sensor for muscle contraction [34], or NaX, the sodium channel that serves as a salt level sensor in the subfornical organ [35].

The ion selectivity of NALCN is clearly important for its function since the wild-type EEKE NALCN but not a mutated calcium-like EEEE isoform rescues the NALCN mutant phenotype in *Drosophila* [10]. How NALCN with its EKEE pore plays a role in rescuing the fly mutant phenotype doesn't appear simply as a replacement of a missing sodium leak conductance though. The fly NALCN mutant is associated with an increased outward potassium current, without altering an inward cation current [10]. There was significant evolutionary pressure to retain the ancestral state with a Ca^2+^ channel-like, NALCN-EEEE pore in many invertebrates, which could serve as a potential calcium leak conductance current reported in invertebrates [36], and at least one chordate (amphioxus) appears to possess a NALCN channel with only an EEEE pore, which may not generate Na^+^ leak currents at all, according to current physiological models.

### Gd3^+^ is not a specific blocker of NALCN currents

10 µm Gd^3+^ has been ascribed as a specific blocker of NALCN currents [4], but this cation also rapidly improves membrane seals, and readily prevents non-specific leak conductances during whole cell recordings of control HEK-293T cells, that in our hands, is indistinguishable from what we observe when we record NALCN channels transfected in HEK-293T cells. 1 mM verapamil, 1 mM Cd^2+^ and 1 mM Co^2+^ also block the Gd^3+^ - sensitive, non-specific leak currents in control HEK-293T cells that have been reported to be characteristics of NALCN expressed currents [4]. NALCN is not natively expressed in HEK-293T cells, and can be detected as expressed protein after standard transient transfection of NALCN containing plasmids in HEK-293T cells. However, we cannot affirm NALCN characterized as a non-specific, voltage-independent, cationic leak conductance *in vitro* [4].

### Future models of NALCN physiology will have to explain NALCN's variable selectivity filters

Mammals lacking NALCN have a disrupted respiratory rhythm [4] and NALCN is also required in rhythmically-active neurons in invertebrates, *Drosophila* and *C. elegans* [9]–[11], [13]. A reliable consequence of NALCN knockdown is a membrane hyperpolarization [4], [11], [37]. Invertebrates have increased sensitivity to volatile anesthetics [38], [39] and defects in exocytosis [[7]–[10], that could relate to NALCN's influence on membrane potential serving to regulate secretion in neurons that require fast or continuous vesicle turnover. Future models will have to justify the highly intriguing and counter-intuitive finding, that the solitary NALCN gene can have a highly variable selectivity filter which resembles a calcium channel (NALCN-EEEE) or a sodium channel (EKEE/NALCN-EEKE). Many invertebrates have alternative selectivity filters, with unique expression patterns in different tissues that appear to suggest that the two different spliced variants of NALCNs are associated with different physiological roles.

## Materials and Methods

### Source of animals

Giant pond snails, *Lymnaea stagnalis* were raised in-house in a snail vivarium and breeding facility in B1-177, Department of Biology, University of Waterloo.

### Sequencing the Lymnaea NALCN cDNA

Two preliminary non-overlapping sequences, spanning a large portion of the NALCN channel coding sequence, had previously been deposited into GenBank (Accession numbers AF484086 and AF484085) [40]. 5′ RACE was used to determine the missing N-terminal coding sequence. Briefly, total RNA was extracted from isolated central nervous system ganglia (CNS) and whole animals using Tri-reagent (Sigma) [41], and reverse transcription was carried out using 1 µg of each RNA extract diluted to 9 µL in water, plus the following reagents: 5 µL of 5× Moloney Murine Leukemia Virus (M-MLV) reverse transcriptase (RTase) buffer (Promega), 2 µL of 10 mM dNTP mix (Fermentas), 0.4 µL of 100 µM LNALCN NT primer (Sigma-Genosys) (**Table S1**), 1 µL of RiboLock RNase inhibitor (Fermentas), 2.6 µL of water, and 1 µL of M-MLV RTase enzyme. cDNA synthesis was carried out at 37°C for 1 hour, and products were co-precipitated with 2 µL of 20 mg/mL glycogen (Fermentas) in ethanol, washed with 70% ethanol, and resuspended with 10 µL of water. 5′ poly-A tailing of cDNAs was achieved by adding the following reagents to the cDNA samples (all from Fermentas): 4 µL of 5× Terminal Deoxynucleotidyl Transferase (TdT) buffer, 4 µL of 1 mM dATP, 1 µL of water, and 1 µL of TdT enzyme. Reactions were carried out at 37°C for 15 minutes then heat-inactivated at 80°C for 3 minutes. Nested PCR was performed to amplify the NT coding sequence and UTR of *Lymnaea* NALCN from poly-A tailed CNS and whole animal cDNA, using nested primer pairs RACE-For1 plus LNALCN-Rev1, and RACE-For2 plus LNALCN-Rev2 (Sigma-Genosys) (**Table S1**). For PCRs, used 2.5 µL of 10× High Fidelity PCR Buffer (Fermentas), 1.5 µL of 25 mM MgCl_2_ (Fermentas), 0.5 µL of 10 mM dNTP mix (Fermentas), 1.25 µL of each primer, 17.38 µL of water, 0.13 µL of High Fidelity PCR Enzyme Mix (Fermentas), and 0.5 µL of tailed cDNA (for 1° PCR reaction) or 1° PCR (for 2° PCR). RACE-amplified DNA from CNS and whole animal cDNAs was cloned into pGEM-T Easy (Promega) and sequenced; both were found to contain the snail NALCN start codon and N-terminal sequence, as well as the 5′UTR. To determine the unknown sequence between the previously deposited AF484086 and AF484085 GenBank sequences, nested PCR spanning the gap was carried out using *Lymnaea* λ-ZAP cDNA libraries made from CNS as template, and primer pairs LNALCN gap 5′1 plus LNALCN gap 3′1 and LNALCN gap 5′2 plus LNALCN gap 3′2 (**Table S1**) as previously described [42]. DNA fragments from PCRs were gel-purified, cloned into pGEM-T Easy, and sequenced as indicated above.

### Phylogenetic analyses of NALCN

NALCN orthologs were gathered by BLAST data-mining of available genomic databases NCBI (Bethesda, MD), Joint Genome Institute, Department of Energy and University of California (DOE-JGI), Washington University in St. Louis (Genome Institute at WUSTL), Baylor College (HGSC), Broad Institute of MIT and Harvard. Sequences were aligned using MUSCLE and evolutionary trees were inferred by maximum parsimony (PAUP 4.0, Swofford) and maximum likelihood (PAML4, Yang).

### Consensus sequencing of Lymnaea NALCN cDNAs

Primers were designed to PCR-amplify the snail NALCN coding sequence in 5 separate, overlapping fragments, using the following nested primer pairs (listed in order from most N-terminal to most C-terminal along the cDNA sequence): LNALCN R1, LNALCN R2, LNALCN R3, LNALCN R4, and LNALCN R5 (**Table S1**). All PCR were carried out as indicated above for the screening of λ-ZAP cDNA libraries, using as templates either the λ-ZAP cDNA library fractions, a CNS cDNA library prepared using a random hexamer primer (**Table S1**), or cDNA libraries generated with NALCN-specific primers. All PCR products were cloned into pGEM-T Easy as indicated above for sequencing, and a minimum of three independent sequences for each position along the transcript were used to build the consensus for both domain II pore isoforms of *Lymnaea* NALCN NALCN-EKEE (GenBank: GJQ806355) and NALCN-EEEE (GenBank: JQ806356). Sequencing of snail NALCN cDNAs confirmed the existence of two mutually exclusive splice variants with variable coding sequences for domain II pore regions of the putative channels.

### Cloning of the Lymnaea NALCN cDNAs into mammalian expression vector pIRES2-DsRed2

*Lymnaea* NALCN splice variants (i.e. EKEE and EEEE) were each cloned into the bicistronic vector pIRES2-DsRed2 (Clontech) in three PCR-amplified fragments. Briefly, two cDNA fragments that were previously cloned into pGEM-T Easy for consensus sequencing of snail NALCN (see above), were combined into a large 2925 bp fragment corresponding to the invariable C-terminal coding sequence of snail NALCN. These were joined by inserting a *Hind*III-*Sac*I-digested insert DNA fragment of the LNALCN R5 subclone into the same restriction enzyme sites in the LNALCN R4 subclone. This assembled DNA was then cloned into the pIRES2- DsRed2 vector via *BamH*I sites flanking the insert. Large N-terminal portions of the two NALCN splice variants (∼3500 bp) were then PCR-amplified from adult CNS made using a NALCN-specific primer (LNALCN-RT 3′) (**Table S1**). Nested PCR pairs (LNALCN S1 5′ 1 plus LNALCN S1 3′, and LNALCN S1 5′ 2 plus LNALCN S1 3′) (**Table S1**) allowed for direct cloning of the PCR product into the pIRES2-DsRed2 harbouring the C-terminal portion of the channel via *Xho*I and *Sal*I restriction enzyme sites. Clones were fully sequenced to confirm the presence of both domain II splice variants and lack of mutations, and these were transfected into HEK-293T cells to confirm expression of DsRed2 from the internal ribosome entry site located downstream of but on the same transcript as the NALCN insert cDNAs. For N-terminal fusions with EGFP, cloned LNALCN cDNAs were excised from the corresponding pIRES2 vectors and inserted into the pEGFP-C1 vector (Clontech) via *Xho*I and *Apa*I.

After our initial cloning of the *Lymnaea* NALCN variants, we identified a potential missense polymorphism in the cloned cDNAs in a region common to both EKEE- and NALCN-EEEE (R1189Q in EKEE; guanosine to adenine substitution). To produce both the R and Q variants of the *Lymnaea* NALCN isoforms, we performed site-directed mutagenesis using the QuikChange protocol (Agilent Technologies) with primers LNALCN QC MF 5′ and LNALCN QC MF 3′ (**Table S1**), as per manufacturer's instructions. The EKEE- and NALCN-EEEE clones containing an arginine (R) at position 1189 were used for **Figure 7**, and the clones with a glutamine (Q) in this position were used for **Figure 8**.

### Cloning of human Unc-80

Primers pairs were designed to amplify the entire coding region of the human Unc-80 cDNA in 4 partial fragments designated PCR-H1 to PCR-H4 (**Table S1**). Reverse transcription was performed using 5 µg of total RNA from human brain (Clontech) with Superscript III reverse transcriptase (Invitrogen) and 50 pmoles of random hexamers (Invitrogen), according to the manufacturer. PCR was performed using a mix of 2.5 Units of Taq DNA Polymerase (Invitrogen) and 2.5 Units of Pfu Turbo DNA Polymerase (Stratagene) with 50 pmoles of each primer, 2 µL of the reverse transcription product, 0.2 mM of equimolar dNTP mix, buffer (20 mM Tris-HCl pH 8.4, 50 mM KCl), in a final volume of 50 µL. The PCR fragments were cloned in the cloning vector pCR2.1 using the TA cloning kit (Invitrogen) and several clones were sequenced on both strands. The Unc-80 cDNA was constructed using unique restriction sites and subsequently subcloned into the mammalian expression vector pIRES2-EGFP (Clontech).

### Transfections and electrophysiological recordings of snail NALCN

Mammalian cells (HEK-293T) were cultured as previously described [43]. For transfection of snail NALCN, fully confluent cells in a 6 mL vented flask were detached using warm Trypsin (Sigma-Aldrich) and split 1∶4 into 35 mm culture dishes containing Dulbecco's Modified Eagle's Medium (DMEM) supplemented with 10% fetal bovine serum (FBS; Sigma) and 100 µM sodium pyruvate (Sigma-Aldrich). After over-night incubation at 37°C to permit cell adhesion and recovery, transfections were prepared by combining 10 µL of Lipofectamine 2000 (Invitrogen), 1 µg of either SNAIL NALCN in pIRES2-DsRed2 or pIRES2-DsRed2 as control (Promega), 1 µg of human Unc-80 in pIRES2-EGFP, and 1 µg of constitutively active SRC kinase SRC Y529F in a pUSEamp vector (kindly provided by Dr. Dejian Ren) in 1.5 mL of OptiMEM (Sigma-Aldrich). Reagents were then incubated for 20 minutes, applied to the cells dropwise, and cells were incubated at 37°C for 4–6 hours, washed 1× with warm supplemented DMEM lacking antibiotics, and incubated overnight at 37°C in the same media used for the wash. The next day, the media was replaced with the same but also containing penicillin/streptomycin (Sigma-Aldrich; as per manufacturer), and cells were transferred to 28°C. After 1–2 days, transfected cells were detached by trypsinization (as above) and plated onto 2 mL round culture dishes containing 2 mL of supplemented DMEM and incubated at 37°C for 1–3 hours to allow cells to attach to the substrate and recover prior to electrophysiological recording.

Whole-cell patch clamp technique was performed as reported previously [26], [42]–[45] using as external bath solution (in mM; all chemicals from Sigma): 150 NaCl, 3.5 KCl, 1 MgCl_2_, 1.2 CaCl_2_, 20 glucose, and 10 HEPES (pH 7.4 with NaOH) with a measured osmolarity of ∼320 mOsm/L. For replacement of external sodium, 150 mM NaCl was replaced with 150 mM NMDG^+^, where the pH was adjusted with HCl and the osmolarity was also measured at ∼320 mOsm/L. Gadolinium (Gd^3+^) was freshly diluted to 10 µM in 150 mM sodium external just prior all experiments. The internal solution contained (in mM): 150 Cs^+^, 120 MES, 10 NaCl, 10 EGTA, 4 CaCl_2_, 0.3 Na_2_GTP, 2 Mg-ATP, and 10 HEPES pH 7.4 with CsOH (∼300 mOsm/L). Recordings were done at room temperature, with patch pipettes bearing resistances between 2 to 5 MΩ, and patches had typical access resistances between 4 to 6 MΩ. Series resistance was compensated to 70% (prediction and correction; 10-µs time lag).

### Western Blotting

EGFP-LNALCN and pEGFP-C1 transfected HEK-293T cells were directly lysed in 2× sample buffer (10% glycerol (v/v), 1% SDS, 50 mM DTT and 62.5 mM Tris pH 6.8), and 25 µL aliquots of each protein sample were separated on 7.5% SDS-PAGE gels. Proteins were transferred to a 0.45 µm PROTRAN® nitrocellulose membrane (Whatman®) and blocked in Tween-20 Tris-buffered saline (TTBS) containing 5% skim milk powder (w/v). The membrane was then incubated with α-GFP antibody (Amsbio; 1∶2000 in TTBS with 5% milk) overnight a 4°C, washed the next day 2×15 minutes with TTBS, then incubated with 1∶1500 goat α-rabbit HRP (Jackson ImmunoResearch Laboratories, Inc.) at room temperature for 3 hours. Membrane was subsequently washed 3×15 min with TTBS, HRP-activated chemiluminescence was detected using the Super Signal West Pico Chemiluminescent system (Pierce Chemical).

### Immunocytochemistry of sections of the Lymnaea central nervous system

The snail NALCN polyclonal antibody was generated using 15mer SYRSVDIRKSLQLEE C-terminal peptide sequence of NALCN coupled to KLH, and raised in rabbits. Central nervous systems from snails were dissected and fixed in 1% paraformaldehyde and 1% acetic acid, and embedded in paraffin. Seven µm sections were immunocytochemically stained with LNALCN antiserum with a procedure described previously [46].

### qPCR of Lymnaea tissues

Developmental schedules of *Lymnaea*, as well as the methods used for both qPCR and semi-quantitative RT-PCR have been described previously in detail [26], [42]. Briefly, mRNA for qPCR analyses was extracted from 50–75% and 100% embryos, grouped according to morphological features of embryonic animals within egg capsules [47], and shell length of juvenile vs. adult snails (1–1.5 cm and 2–2.5 cm respectively) [48]. *Lymnaea* transcripts were amplified by quantitative RT-PCR (qPCR) with primers designed against an invariable portion of the snail NALCN cDNA (i.e. LNALCN qPCR UNV 5′ and LNALCN qPCR UNV 3′) (**Table S1**), as well as primers specific for each of the domain II splice isoform (LNALCN DIIK 5′ plus LNALCN DIIK 3′, and LNALCN DIIE 5′ plus LNALCN DIIE 3′ (**Table S1**). qPCR transcripts were normalized against standards, actin, SDHA and HPRT1 (**Table S1**). Cycle threshold (CT) values for the HPRT1 gene produced the lowest stability value (i.e. 0.098) using NormFinder software [49], indicating its suitability as a reference gene. Expression levels of genes/isoforms were normalized relative to HPRT1 using the ratio [50]: (E_target gene_)^ΔCTtarget gene^/(E_HPRT1_)^ΔCTHPRT1^. Amplicons ranged from 102 to 145 bp, producing single products (as determined by melting curve analysis and visualization of electrophoresed qPCR products on ethidium bromide-stained agarose gels), with PCR efficiencies (E) ranging from 89.9 to 100.6% (**Table S1**), using 1∶5 serial dilutions of pooled cDNA from all RNA extracts as template. qPCR reactions were carried out in quadruplicate, and standardized between 96 well plate samples with primers against HPRT1.

### Reverse Northern blotting

For reverse Northern blot analyses, soma-ablated axons adhered to culture dishes were rinsed three times in sterile saline before cultured axons were bathed and lifted from the adhesive substrate by trituration in Trizol reagent (Invitrogen). Subsequent to Trizol extraction, total RNA (200 ng) was amplified by SMART cDNA synthesis (Clontech). 32P-labeled/PCR-amplified cDNA inserts were served as probes on blots spotted with DNA plasmids (200 ng) on a Hybond-N nylon membrane (Amersham Biosciences) coding for DNA fragments of *Lymnaea* calcium channel and NALCN clones (GenBank accession number, corresponding to the amino acid sequences, LCa_v_1 (AF484079 [GenBank], 373–670) [45], [51], [52], LCa_v_2 (AF484082 [GenBank] , 302–621) [44], [53], LCa_v_3 (AF484084 [GenBank], 848–1111) [26], [42], LNALCN (JQ806355 [GenBank], 4525–5285) [40] and subsequently imaged via a PhosphorImager (Bio-Rad).

## Supporting Information

Appendix S1 **Annotated genomic sequences of exons flanking Exon 15 in NALCN channels**. NALCN orthologs spanning exon 15 from different Phyla (Porifera, Placozoa, Cnidaria, Platyhelminthes, Nematoda, Arthropoda, Mollusca, Annelida, Hemichordata, Chordata) were gathered by BLAST data-mining of available genomic databases NCBI (Bethesda, MD), Joint Genome Institute, Department of Energy and University of California (DOE-JGI), Washington University in St. Louis (Genome Institute at WUSTL), Baylor College (HGSC), Broad Institute of MIT and Harvard.
(DOCX)

Appendix S2 **Annotated genomic sequences of exons flanking Exon 31 in NALCN channels.** NALCN orthologs spanning exon 31 from different Phyla (Porifera, Placozoa, Cnidaria, Platyhelminthes, Nematoda, Arthropoda, Mollusca, Annelida, Hemichordata, Chordata)were gathered by BLAST data-mining of available genomic databases NCBI (Bethesda, MD), Joint Genome Institute, Department of Energy and University of California (DOE-JGI), Washington University in St. Louis (Genome Institute at WUSTL), Baylor College (HGSC), Broad Institute of MIT and Harvard.
(DOCX)

Table S1 **DNA primers sequences for Lymnaea NALCN and human UNC-80 used in cDNA synthesis, DNA sequencing and mRNA quantitation (qPCR).**
(DOCX)
