# Supplementary material for: NALCN Ion Channels Have Alternative Selectivity Filters Resembling Calcium Channels or Sodium Channels
Source: PLoS One. 2013 Jan 28;8(1):e55088. doi: 10.1371/journal.pone.0055088 (PMC3557258; doi:10.1371/journal.pone.0055088)

Appendix S2: Annotated genomic sequences of exons flanking Exon 31 in NALCN Channels

Contents

[Phylum Porifera 4](#_Toc317707584)

[*1 - Amphimedon queenslandica* 4](#_Toc317707585)

[Phylum Placozoa 4](#_Toc317707586)

[*2 - Trichoplax adherens* 4](#_Toc317707587)

[Phylum Cnidaria 4](#_Toc317707588)

[Class Hydrozoa 4](#_Toc317707589)

[*3 - Hydra magnipapillata* 4](#_Toc317707590)

[*4 - Acropora digitifera* 5](#_Toc317707591)

[Class Anthozoa 5](#_Toc317707592)

[*5 - Nematostella vectensis* 5](#_Toc317707593)

[Phylum Platyhelminthes 6](#_Toc317707594)

[Class Turbellaria 6](#_Toc317707595)

[*6 - Schmidtea mediterranea* 6](#_Toc317707596)

[Class Trematoda 7](#_Toc317707597)

[*7 – Schistosoma mansoni* 7](#_Toc317707598)

[*8 – Clonorchis sinensis* 7](#_Toc317707599)

[Phylum Nematoda 8](#_Toc317707600)

[Class Enoplea 8](#_Toc317707601)

[*9 – Trichinella spiralis* 8](#_Toc317707602)

[Class Rhabditea 8](#_Toc317707603)

[*10 – Caenorhabditis elegans nca-1* 8](#_Toc317707604)

[*11 – Caenorhabditis elegans nca-2* 8](#_Toc317707605)

[Phylum Arthropoda 9](#_Toc317707606)

[Class Insecta 9](#_Toc317707607)

[*12 – Acyrthosiphon pisum* 9](#_Toc317707608)

[*13 – Drosophila melanogaster* 9](#_Toc317707609)

[Class Crustacea 9](#_Toc317707610)

[*14 – Daphnia magna* 9](#_Toc317707611)

[Class Myriapoda 10](#_Toc317707612)

[*15 – Strigamia maritima* 10](#_Toc317707613)

[Class Arachnida 11](#_Toc317707614)

[Order Parasitiformes 11](#_Toc317707615)

[*16 – Ixodes scapularis* 11](#_Toc317707616)

[*17 – Varroa destructor* 12](#_Toc317707617)

[Order Acariformes 12](#_Toc317707618)

[*18 – Tetranychus urticae* 12](#_Toc317707619)

[Phylum Mollusca 13](#_Toc317707620)

[Class Gastropoda 13](#_Toc317707621)

[Subclass Heterobranchia 13](#_Toc317707622)

[*19 – Biomphalaria glabrata* 13](#_Toc317707623)

[*20 – Aplysia californica* 13](#_Toc317707624)

[Subclass Patellogastropoda 13](#_Toc317707625)

[*21 – Lottia gigantea* 13](#_Toc317707626)

[Phylum Annelida 14](#_Toc317707627)

[Class Polychaeta 14](#_Toc317707628)

[*22 – Capitella teleta* 14](#_Toc317707629)

[Class Clitellata 14](#_Toc317707630)

[*23 – Helobdella robusta* 14](#_Toc317707631)

[Phylum Echinodermata 15](#_Toc317707632)

[Class Echinoidea 15](#_Toc317707633)

[*24 – Strongylocentrotus purpuratus* 15](#_Toc317707634)

[*25 – Lytechinus variegatus* 15](#_Toc317707635)

[Phylum Hemichordata 16](#_Toc317707636)

[*26 – Saccoglossus kowalevskii* 16](#_Toc317707637)

[Phylum Chordata 16](#_Toc317707638)

[Subphylum Urochordata 16](#_Toc317707639)

[*27 – Ciona intestinalis* 16](#_Toc317707640)

[Subphylum Cephalochordata 17](#_Toc317707641)

[*28 – Branchiostoma floridae* 17](#_Toc317707642)

[Subphylum Vertebrata 17](#_Toc317707643)

[*29 – Xenopus tropicalis* 17](#_Toc317707644)

[*30 – Takifugu (Fugu) rubripes* 17](#_Toc317707645)

[*31 – Homo sapiens* 17](#_Toc317707646)

# Phylum Porifera

### *1 - Amphimedon queenslandica*

NCBI: scaffold 13303, Contig 4903, 14014-26892


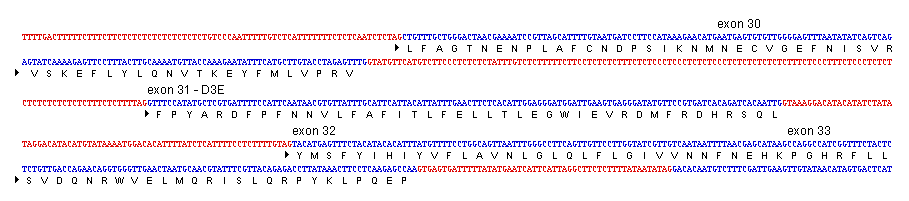


# Phylum Placozoa

### *2 - Trichoplax adherens*

JGI: scaffold 1:145803-159107


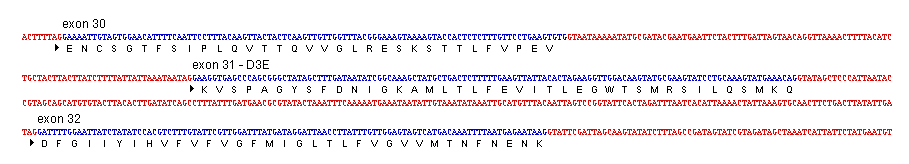


# Phylum Cnidaria

## Class Hydrozoa

### *3 - Hydra magnipapillata*

NCBI: scaffold 30352, Contig 102146, 650-1039; scaffold 3050, Contig 101502 1722-63


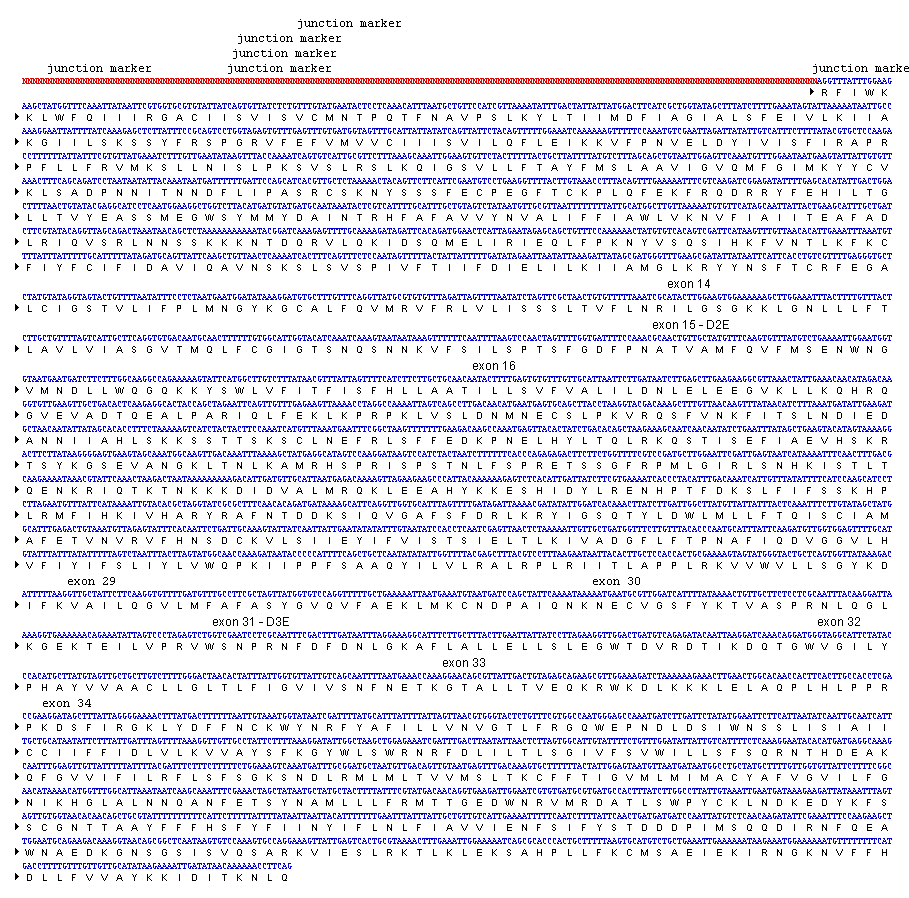


### *4 - Acropora digitifera*

NCBI: Scaffold 45168, contig 2201, 3333- 8600


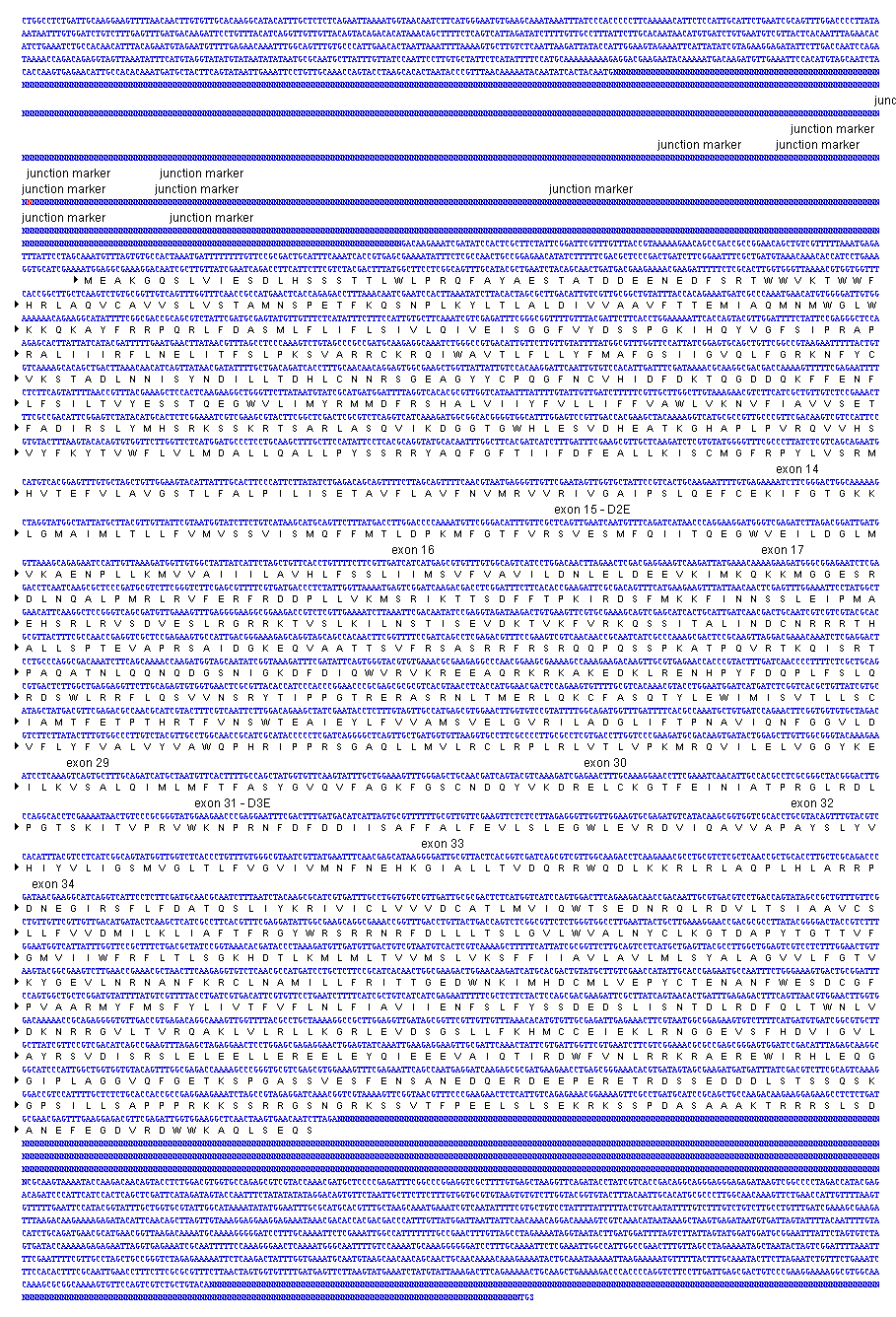


## Class Anthozoa

### *5 - Nematostella vectensis*

NCBI: scaffold 32, Cont5684, 7634- 12910


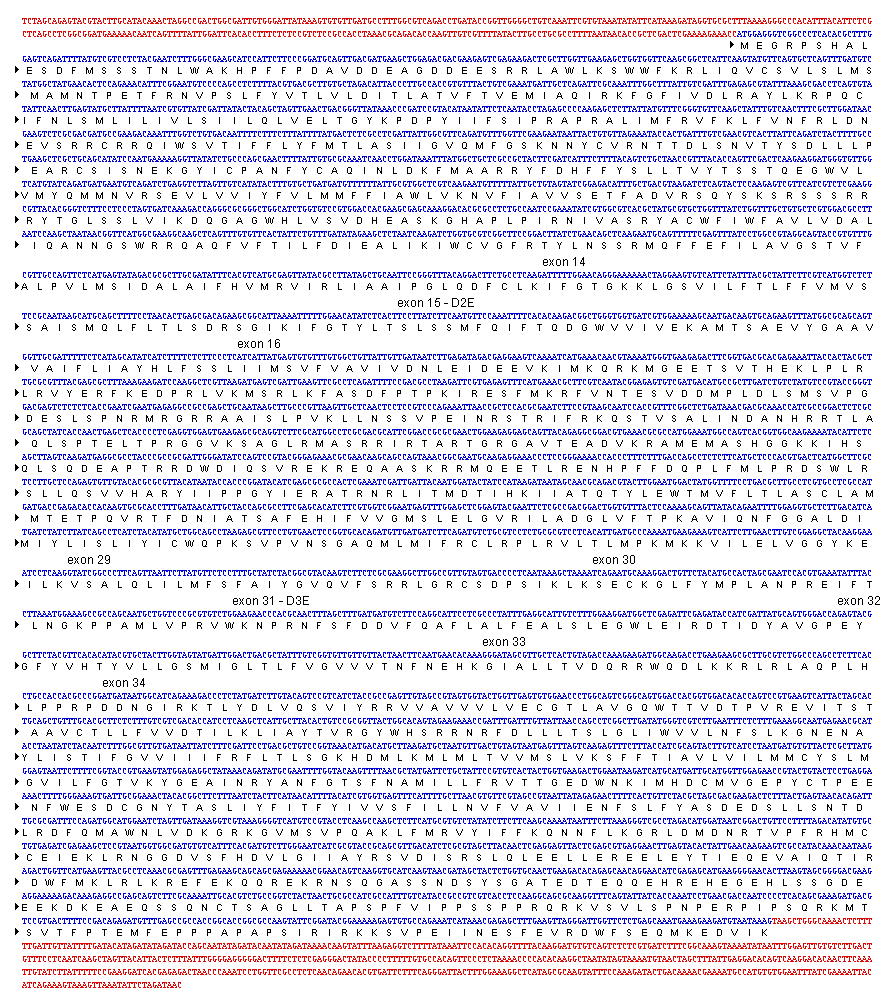


# Phylum Platyhelminthes

## Class Turbellaria

### *6 - Schmidtea mediterranea*

NCBI: v3.1, Contig 8353.3 8092- 18711; Contig 6686.1,467-29539; Contig 13405.2 18-11821


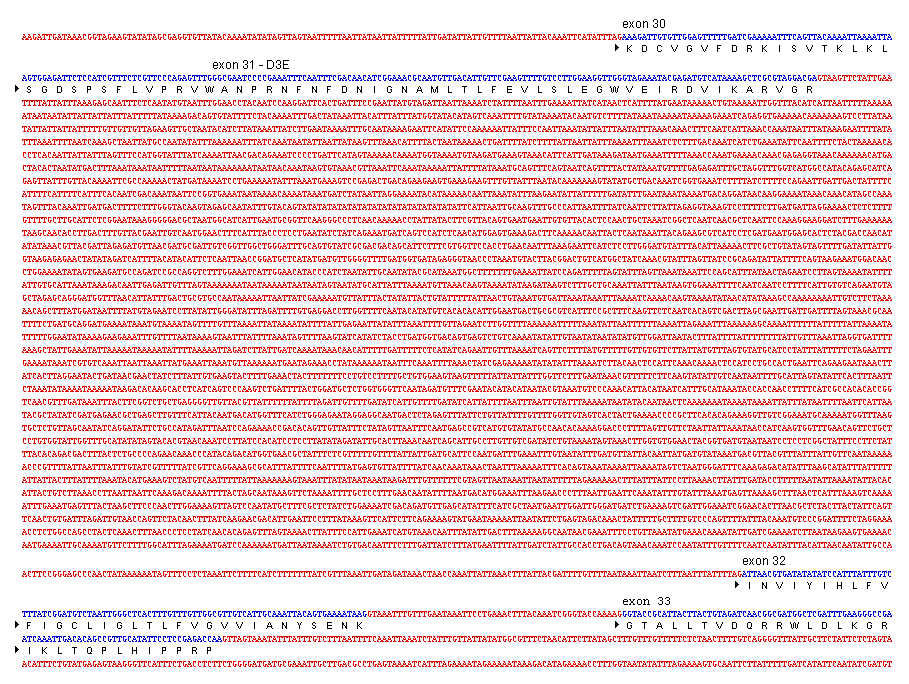


## Class Trematoda

### *7 – Schistosoma mansoni*

NCBI: Smp 141780, scaffold 003392, 117548193-117659558


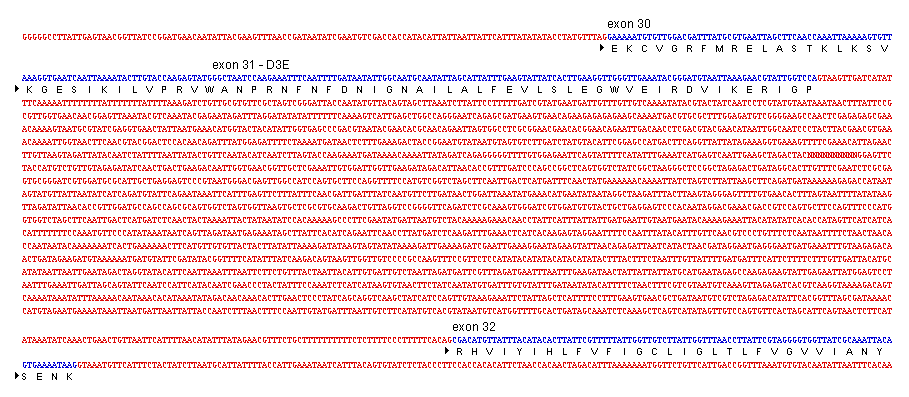


### *8 – Clonorchis sinensis*

NCBI: v2.0 contig 2993, 179514-258411


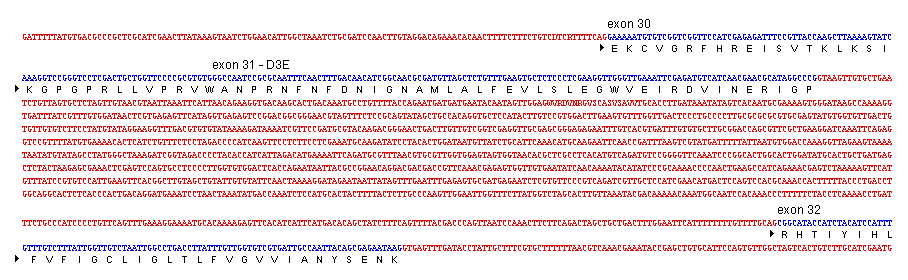


# Phylum Nematoda

## Class Enoplea

### *9 – Trichinella spiralis*

NCBI: Contig 6.27, 129954-143311


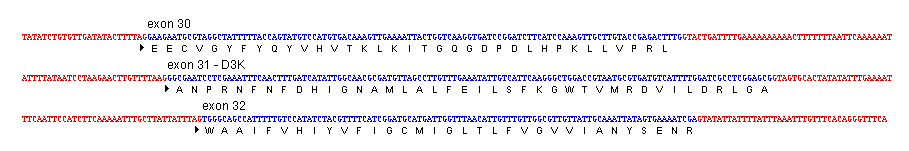


## Class Rhabditea

### *10 – Caenorhabditis elegans nca-1*

NCBI: Chromosome 4: 6157296-6173024


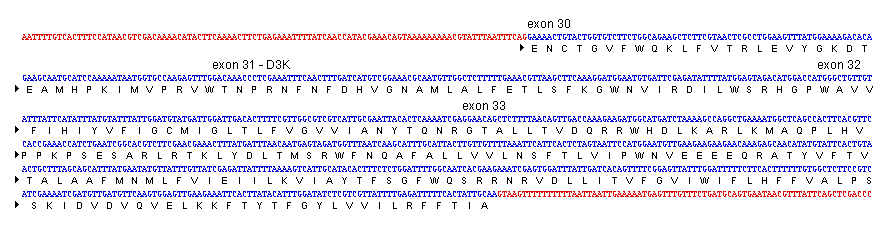


### *11 – Caenorhabditis elegans nca-2*

NCBI: Chromosome 3: 4965623-4979692


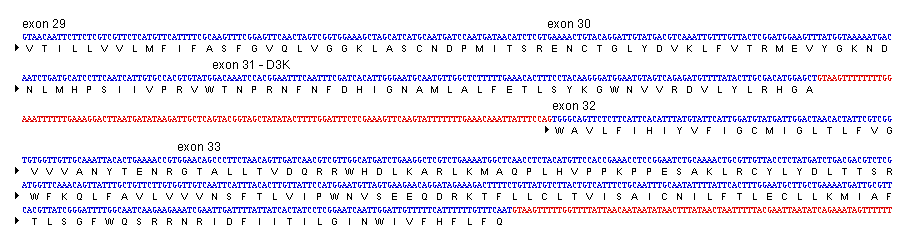


# Phylum Arthropoda

## Class Insecta

### *12 – Acyrthosiphon pisum*

NCBI: Contig 29447: 3302- 1; Contig 29451: 18686-21952


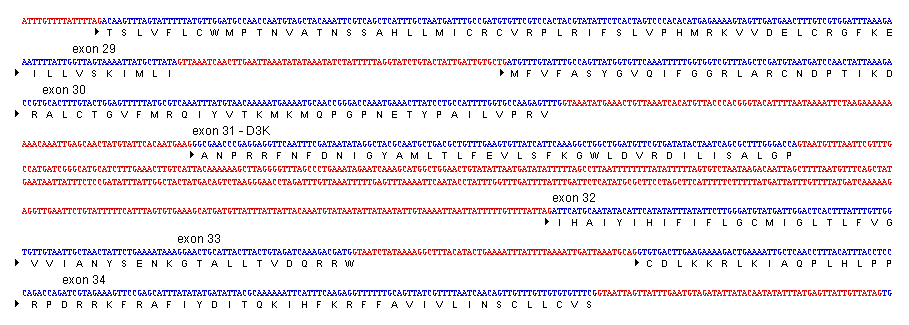


### *13 – Drosophila melanogaster*

NCBI: Chromosome X, 14160400-14171207


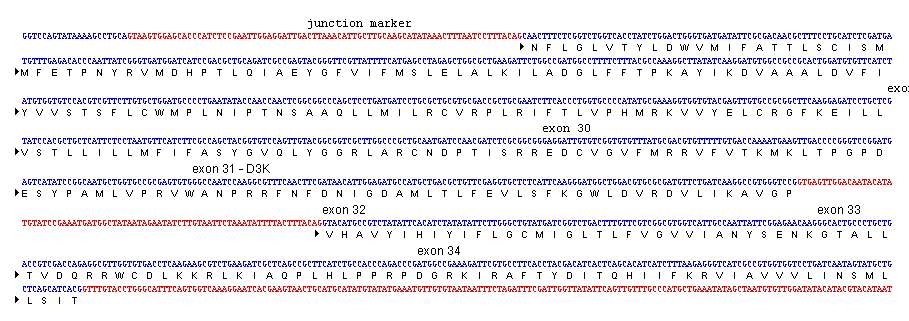


## Class Crustacea

### *14 – Daphnia magna*

NCBI: Scaffold 58, Contig 3121: 60375-67067


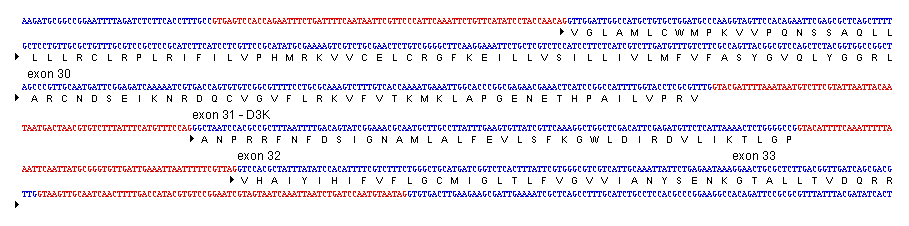


## Class Myriapoda

### *15 – Strigamia maritima*

NCBI: Contig 7180001228033, 48658 - 56466


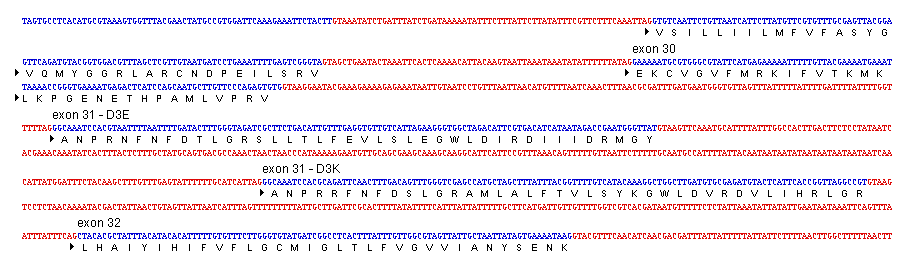


## Class Arachnida

### Order Parasitiformes

### *16 – Ixodes scapularis*

NCBI: Contig 1108378808160, 172-537; Contig 1107729028268, 167-5029; Contig 1107728763546, 561-926; Contig 1107729145460, 288-20352; Contig 1108378222572, 2917-3909


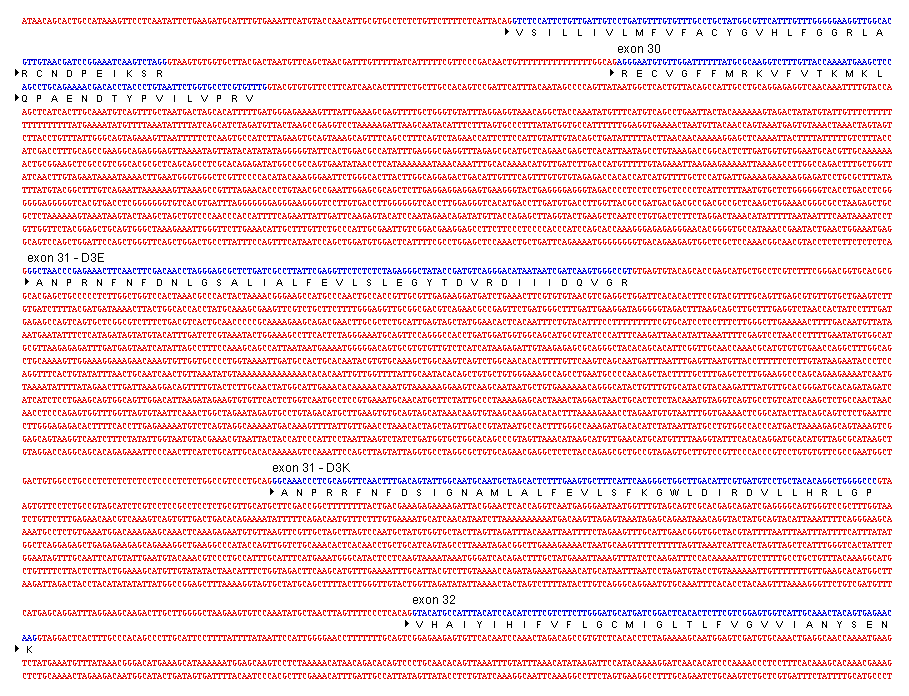


### *17 – Varroa destructor*

NCBI: VDK00036756-2411 507-623; VDK00059420-1771, 1629-1769; VDK00030335-2668 936-2001; VDK00000562-7992 46-5915


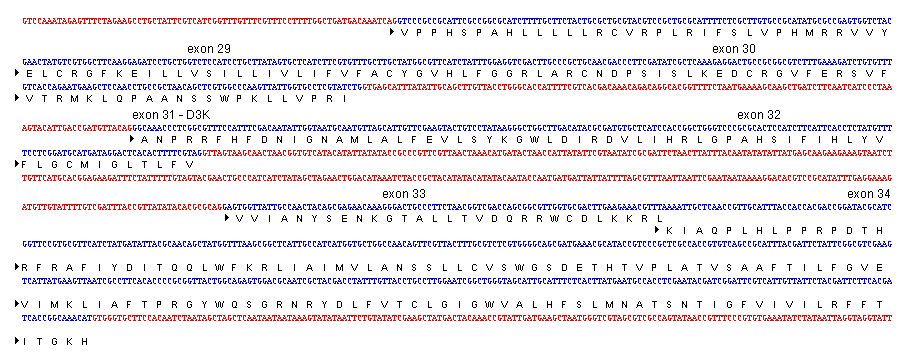


### Order Acariformes

### *18 – Tetranychus urticae*

NCBI: CAEY00000000 data, contig scaffold 15.5, 286455- 293662


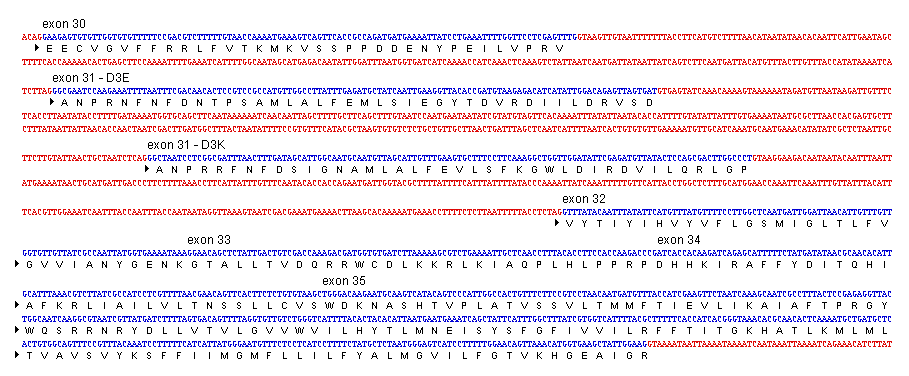


# Phylum Mollusca

## Class Gastropoda

### Subclass Heterobranchia

### *19 – Biomphalaria glabrata*

WUSTL: Scaffold 145 Contig 441684, 87044-118030


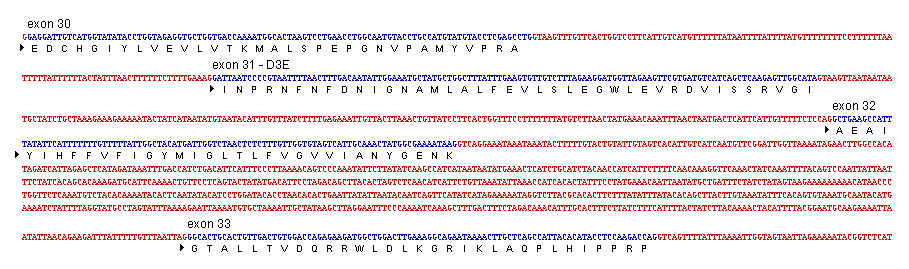


### *20 – Aplysia californica*

NCBI: Contig 2.50281, 1773-27969; Contig 2.50280 1273-21987; Contig 2.50279 11656-17431


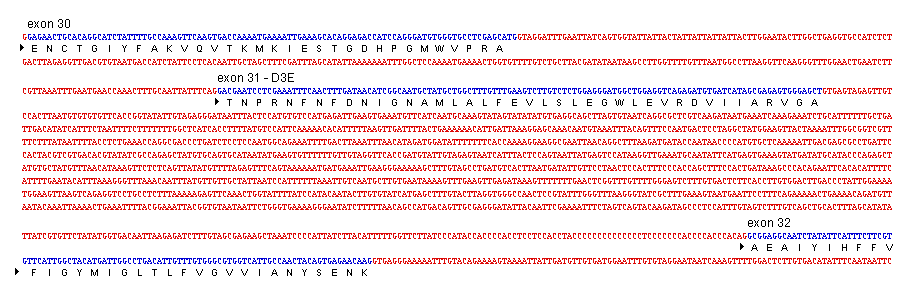


### Subclass Patellogastropoda

### *21 – Lottia gigantea*

JGI: contig scaffold 155, 104459-131590


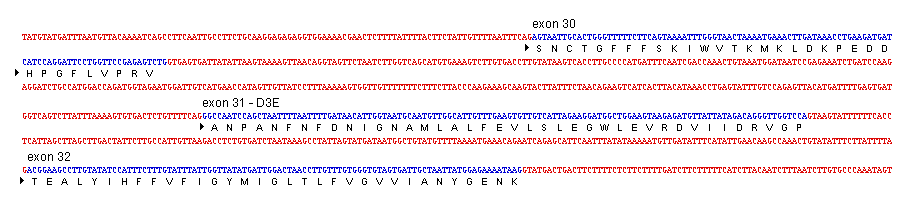


# Phylum Annelida

## Class Polychaeta

### *22 – Capitella teleta*

JGI: contig scaffold 25, 212214-220222


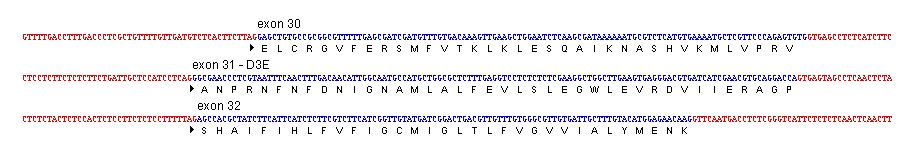


## Class Clitellata

### *23 – Helobdella robusta*

JGI: contig scaffold 5, 289590-301844


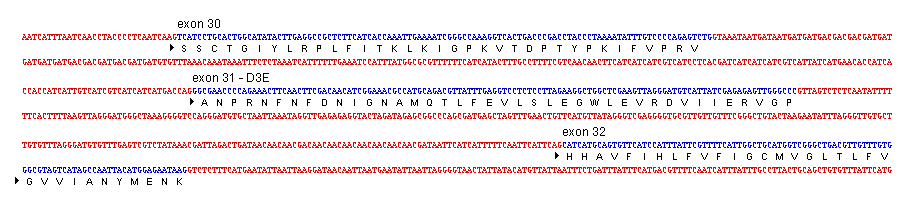


# Phylum Echinodermata

## Class Echinoidea

### *24 – Strongylocentrotus purpuratus*

NCBI: Contig 96934, 414-491; Contig 96933, 2965-7022; Contig 96932, 8139-14537; Contig 96931, 3137-22726; Contig 96929, 1131-3588


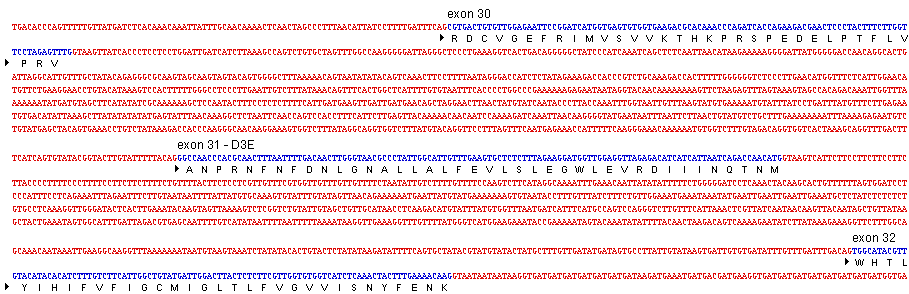


### *25 – Lytechinus variegatus*

NCBI: Contig 480969, 1846-6401; Contig 480968, 3359-7203; Contig 204961, 171-350; Contig 480966, 2297-17986; Contig 480965, 194-307; Contig 480963, 1437-1614; Contig 482258, 3-152; Contig 480962, 562-7143; Contig 480961, 771-10451


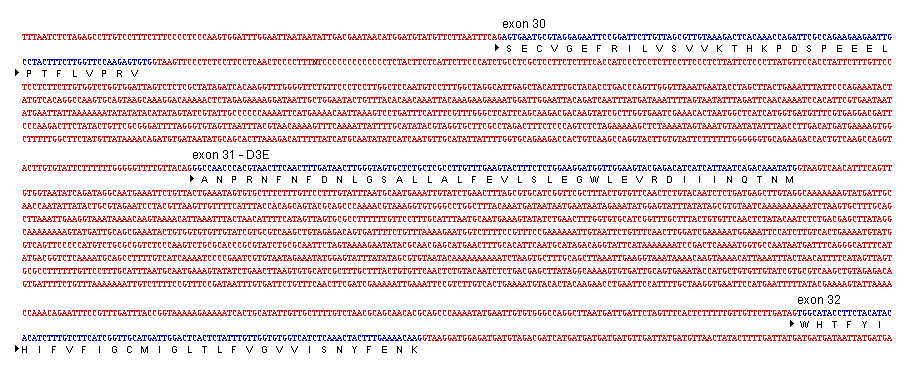


# Phylum Hemichordata

### *26 – Saccoglossus kowalevskii*

NCBI: Contig 42470, 1414-1698; Contig 94515, 742-2171; Contig 12578, 537-683; Contig 94514, 228-645; Contig 94513, 1137-9244; Contig 4553, 1543-8676; Contig 4552, 1164-1313; Contig 4551, 123-1320; Contig 1071-1226


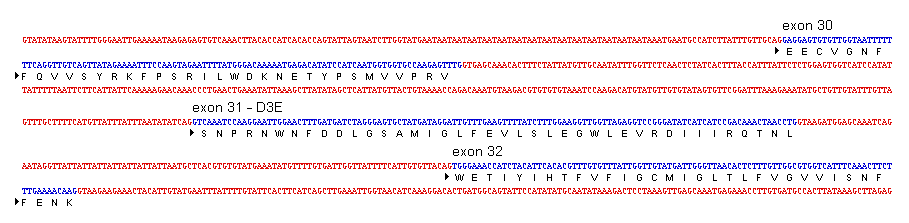


# **Phylum Chordata**

## Subphylum Urochordata

### *27 – Ciona intestinalis*

JGI: Scaffold chr_03q, 4182358- 4212087


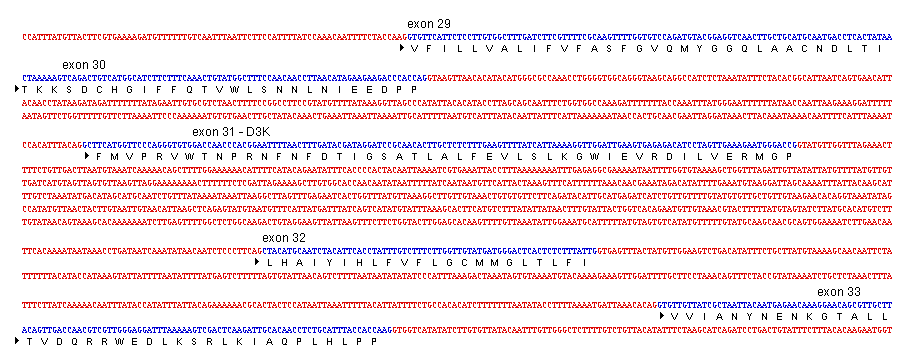


## Subphylum Cephalochordata

### *28 – Branchiostoma floridae*

JGI: scaffold 29, 2388610-2441630


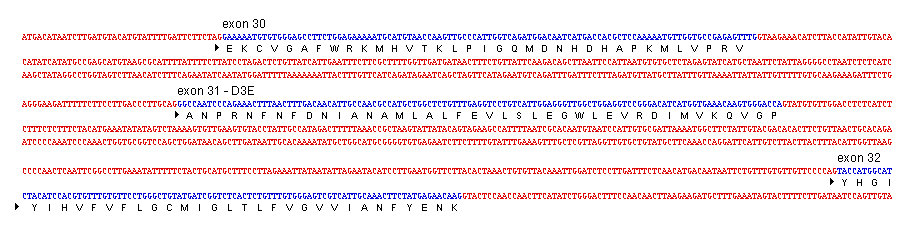


## Subphylum Vertebrata

### *29 – Xenopus tropicalis*

JGI: scaffold 261: 429114-544059


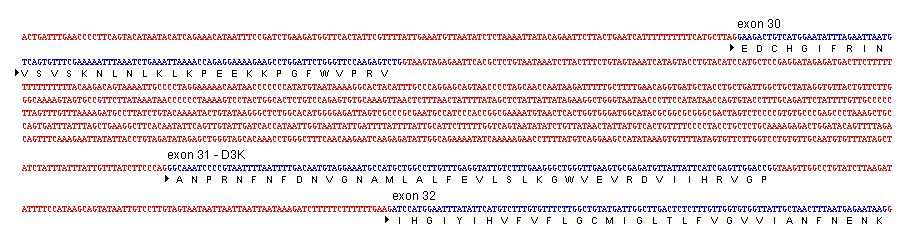


### *30 – Takifugu (Fugu) rubripes*

NCBI: scaffold 31, 661152-693568


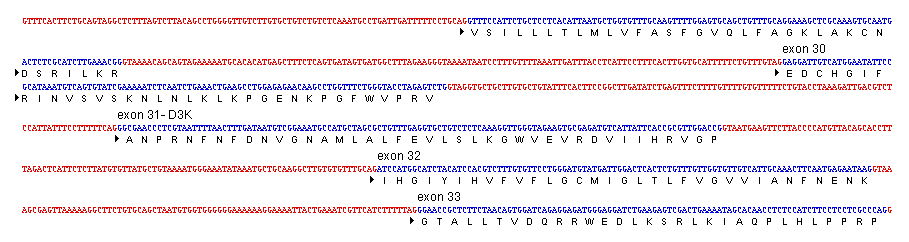


### *31 – Homo sapiens*

NCBI: chromosome 13, GRCh37.p5, 101706130..102068813


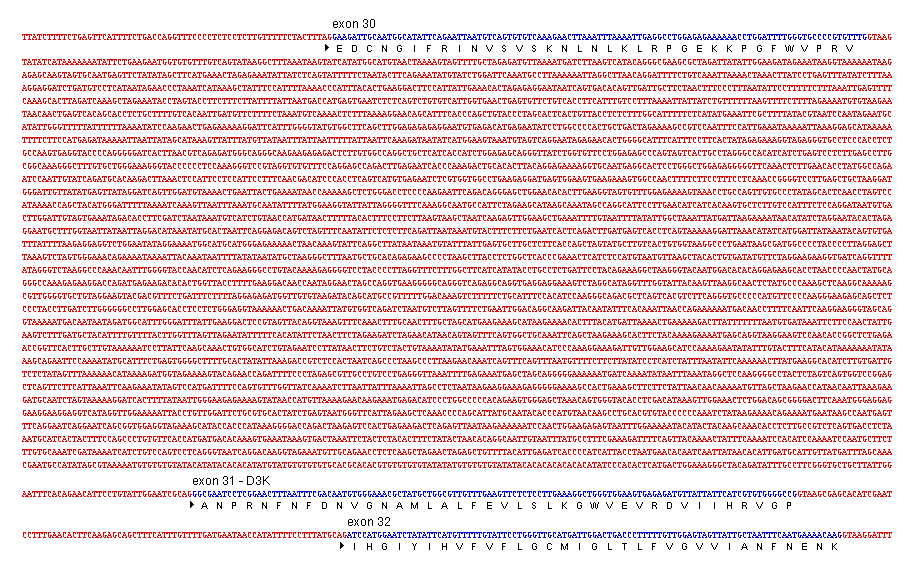

Supplement: Appendix S2 — Annotated genomic sequences of exons flanking Exon 31 in NALCN channels. NALCN orthologs spanning exon 31 from different Phyla (Porifera, Placozoa, Cnidaria, Platyhelminthes, Nematoda, Arthropoda, Mollusca, Annelida, Hemichordata, Chordata)were gathered by BLAST data-mining of available genomic databases NCBI (Bethesda, MD), Joint Genome Institute, Department of Energy and University of California (DOE-JGI), Washington University in St. Louis (Genome Institute at WUSTL), Baylor College (HGSC), Broad Institute of MIT and Harvard. (DOCX) [file pone.0055088.s002.docx]
