# Supplementary material for: NALCN Ion Channels Have Alternative Selectivity Filters Resembling Calcium Channels or Sodium Channels
Source: PLoS One. 2013 Jan 28;8(1):e55088. doi: 10.1371/journal.pone.0055088 (PMC3557258; doi:10.1371/journal.pone.0055088)
Supplement: Table S1 — DNA primers sequences for Lymnaea NALCN and human UNC-80 used in cDNA synthesis, DNA sequencing and mRNA quantitation (qPCR). (DOCX) [file pone.0055088.s003.docx]

Table S1. Table S1. DNA primers sequences for Lymnaea NALCN and human UNC-80 used in cDNA synthesis, DNA sequencing and mRNA quantitation (qPCR)
